# Deubiquitinating enzymes: Key regulators of ferroptosis and pyroptosis and novel targets for cancer intervention

**DOI:** 10.7150/ijbs.111867

**Published:** 2025-06-09

**Authors:** Sheng-Kai Hsu, I-Ying Kuo, Pin-Yuan Lin, Chon-Kit Chou, Ching-Chung Ko, Wen-Tsan Chang, Chien-Chih Chiu

**Affiliations:** 1 Department of Biotechnology, Kaohsiung Medical University, Kaohsiung 807, Taiwan.; 2 Institute of Chinese Medical Sciences, University of Macau, Macau SAR, China.; 3 Department of Medical Imaging, Chi Mei Medical Center, Tainan 710, Taiwan.; 4 Department of Health and Nutrition, Chia Nan University of Pharmacy and Science, Tainan 717, Taiwan.; 5 Division of General and Digestive Surgery, Department of Surgery, Kaohsiung Medical University Hospital, Kaohsiung 807, Taiwan.; 6 Department of Surgery, School of Medicine, College of Medicine, Kaohsiung Medical University, Kaohsiung 807, Taiwan.; 7 Organ Transplantation Center, Kaohsiung Medical University Hospital, Kaohsiung, 807, Taiwan.; 8 Department of Biological Sciences, National Sun Yat-Sen University, Kaohsiung 804, Taiwan.; 9 Center for Cancer Research, Kaohsiung Medical University Hospital, Kaohsiung Medical University, Kaohsiung 807, Taiwan.; 10 Department of Medical Research, Kaohsiung Medical University Hospital, Kaohsiung 807, Taiwan.; 11 Department of Medical Laboratory Science and Biotechnology, Kaohsiung Medical University, Kaohsiung 807, Taiwan.

**Keywords:** Deubiquitinating enzymes (DUBs), posttranslational modification (PTM), ferroptosis, pyroptosis, chemosensitivity, antitumor immunity

## Abstract

Most chemotherapeutic drugs are introduced to eliminate tumors by targeting apoptotic cell death, but chemoresistance frequently develops owing to the aberrant apoptotic machinery. Although the emergence of immune checkpoint blockade (ICB) has revolutionized cancer therapeutics, poor responses to ICB and an immunosuppressive tumor microenvironment are commonly observed in solid tumors. Hence, restoring chemosensitivity and immunosurveillance is important for improving patient outcomes. Recently, the induction of nonapoptotic programmed cell death (PCD), such as ferroptosis and pyroptosis, has received much attention since these alternative forms of cell death potentially increase chemosensitivity and augment antitumor immunity. Ubiquitination and deubiquitination are well-recognized posttranslational modifications, and the balance between these processes is important for maintaining cellular homeostasis. Dysregulation of deubiquitinating enzymes (DUBs) is reportedly associated with tumor progression. Additionally, emerging studies have suggested the involvement of DUBs in modulating cellular susceptibility to nonapoptotic PCD. Nevertheless, the crosstalk between DUBs and nonapoptotic PCDs and their implications for cancer treatment have not been thoroughly reviewed. In this review, we elucidate the roles of DUBs in regulating ferroptosis and pyroptosis via their DUB activities or noncanonical functions. Moreover, we thoroughly discuss the challenges and urgent problems associated with targeting DUBs to induce nonapoptotic PCDs as cancer therapeutics.

## Introduction

In recent decades, the biological characteristics and molecular mechanism of apoptosis have been well investigated; furthermore, cancer chemotherapeutics (e.g., cisplatin, gemcitabine, and 5-fluorouracil (5-FU)) have been shown to trigger apoptosis and eliminate tumors 1. However, drug resistance presents a major challenge for cancer management, and apoptosis dysfunction is a critical factor contributing to intrinsic or acquired chemoresistance 2. For example, following receiving gemcitabine treatment, pancreatic cancer patients with high expression of the antiapoptotic protein Bcl-xL exhibit poorer overall survival and recurrence-free survival than their counterparts do. 3. Moreover, increased Bcl-xL levels were also observed, particularly in recurrent epithelial ovarian tumors following platinum-based chemotherapies 4.

Recently, the emergence of immune checkpoint blockade (ICB) has led to positive results, especially for patients with hematological malignancies. Unfortunately, poor response to ICB and immune evasion are observed in solid tumors because of the immunosuppressive tumor microenvironment (TME) 5. Thus, restoring chemosensitivity and immunosurveillance is of paramount importance for improving patient outcomes. In recent years, the induction of nonapoptotic programmed cell death (PCD), such as ferroptosis and pyroptosis, has received much attention since these processes are capable of increasing chemosensitivity and enhancing antitumor immunity 6-8. Liu and colleagues reported that the induction of autophagy-dependent ferroptosis markedly eliminates multidrug-resistant retinoblastoma cells 6. In addition, gasdermin E (GSDME)-mediated pyroptosis was reported to potentiate chemosensitivity and facilitate T-cell infiltration in non-small cell lung cancer (NSCLC) 8. Overall, the initiation of nonapoptotic PCD seems to be a promising strategy for cancer treatment.

Ubiquitination is a well-recognized posttranslational modification (PTM), and its biological process is catalyzed by an enzymatic cascade involving ubiquitin-activating enzyme (E1), ubiquitin-conjugating enzyme (E2), and ubiquitin ligase (E3) 9, 10. Human deubiquitinating enzymes (DUBs) are members of the protein superfamily and reverse ubiquitination by deconjugating ubiquitins from target proteins to increase their stability or regulate cellular signaling 11, 12. DUB subfamilies are classified into two classes, namely, cysteine proteases and metalloproteases, according to their domain conservation and sequence similarity. Ovarian tumor proteases (OTUs), ubiquitin carboxy-terminal hydrolases (UCHs), ubiquitin-specific proteases (USPs), Machado-Joseph domain-containing proteases (MJDs), monocyte chemotactic protein-induced proteins (MCPIPs), motifs interacting with Ub-containing novel DUBs (MINDYs), and Zn-finger and UFSP domain proteins (ZUFSPs) are among the seven subfamilies of cysteine proteases. The only metalloproteases in the family are JAMM/MPN domain-associated metallopeptidases (JAMMs) 13. In general, the balance between ubiquitination and deubiquitination is strictly controlled to maintain cellular homeostasis, but the aberrant expression of DUBs is tightly associated with aggressive features of cancer 14-18. A growing body of research has revealed the involvement of DUBs in determining the cellular susceptibility to ferroptosis and pyroptosis 19-22. Intriguingly, DUBs serve as Jekyll and Hyde in the modulation of nonapoptotic PCD in different cellular contexts 23, 24. Nevertheless, the relationship between DUBs and nonapoptotic PCD has not been comprehensively reviewed.

In this review, we elucidate the roles of DUBs in the modulation of ferroptosis and pyroptosis via distinct molecular mechanisms and their implications for cancer treatment. Furthermore, the limitations and critical problems associated with targeting DUBs to induce nonapoptotic PCD as a cancer therapeutic strategy are also discussed.

## Article I. The role of DUBs in the modulation of ferroptosis

Aberrant apoptotic machinery (e.g., loss of caspases and upregulation of antiapoptotic proteins) plays a critical role in intrinsic and acquired drug resistance 25, 26, making the induction of nonapoptotic PCD a potential strategy for overcoming chemoresistance. Indeed, previous studies have revealed that the induction of ferroptosis is associated with increased chemosensitivity in multiple cancers, such as esophageal squamous cell carcinoma, lung adenocarcinoma, and head and neck cancer 27-29. Moreover, as a proinflammatory type of PCD, the initiation of ferroptosis promotes the infiltration of IFNγ^+^CD8^+^ T cells into tumors and sensitizes cancer cells to anti-PD-L1 agents 30. These findings highlight the potential of ferroptosis in cancer therapeutics by improving chemosensitivity and antitumor immunity.

Ferroptosis is an iron-dependent and caspase-independent PCD process that is morphologically and biochemically distinct from apoptosis. Its morphological features include cytoplasmic swelling, loss of membrane integrity, and especially reduced mitochondrial cristae 31. Biochemically, ferroptosis is characterized by the accumulation of intracellular iron, increased oxidative stress, and lipid peroxidation 32. Elevated levels of iron facilitate the production of reactive oxygen species (ROS) and increase lipoxygenase activity, which promotes lipid peroxidation. However, several antioxidant defense systems (e.g., the SLC7A11-GSH-GPX4 system, Nrf2 signaling, and FSP1-CoQ10 system) can counteract cellular oxidative stress, thus blocking ferroptosis 31. Altogether, the initiation of ferroptotic cell death is intricately orchestrated by the interplay of these molecular mechanisms. In the following sections, we elucidate the involvement of DUBs in ferroptosis modulation through distinct mechanisms, including the regulation of iron homeostasis, cysteine metabolism, calcium (Ca^2+^) homeostasis, lipid metabolism, and the nuclear factor erythroid 2-related factor 2 (Nrf2) signaling pathway.

### Section 1.01. Iron homeostasis

**Figure 1:**
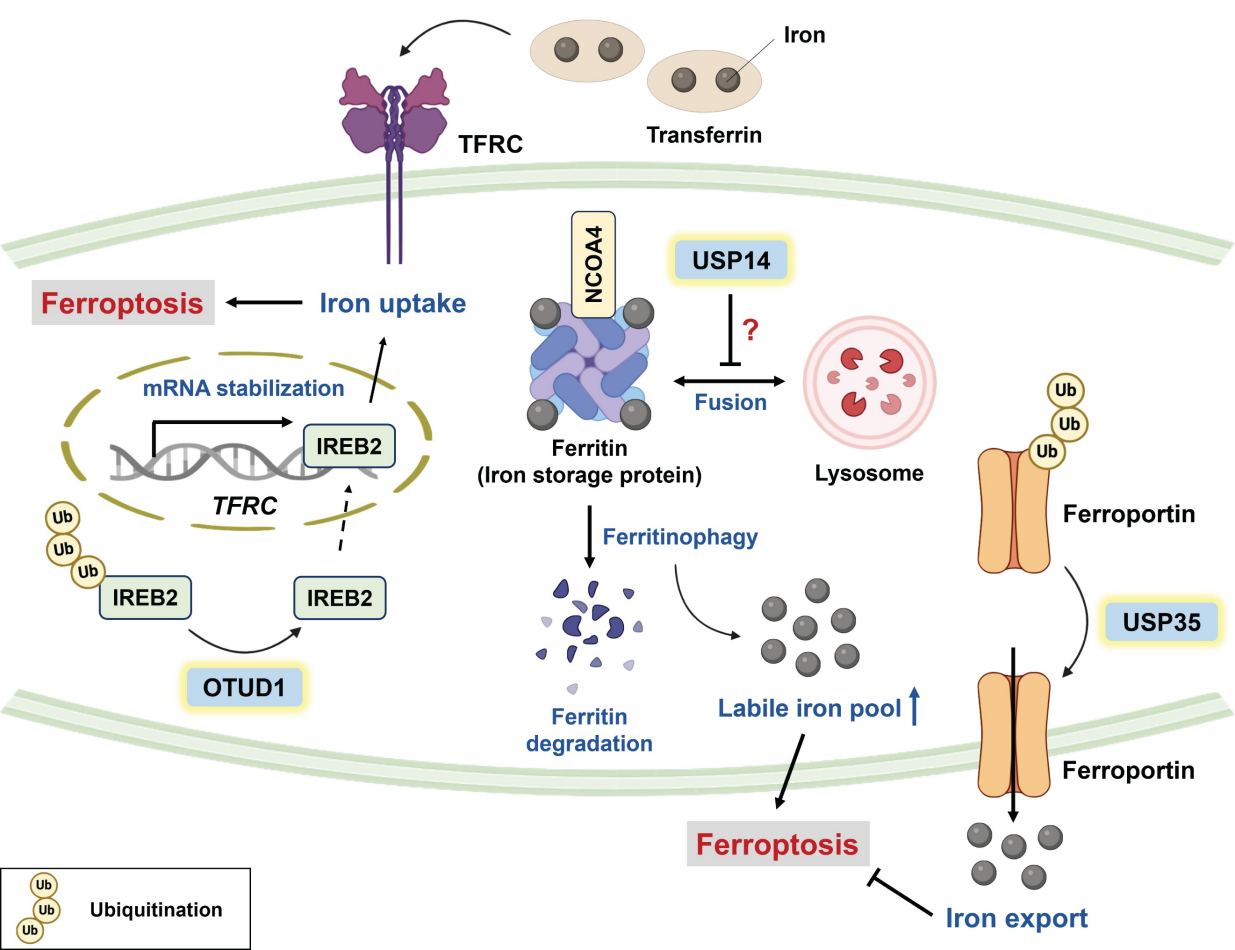
Roles of DUBs in ferroptosis through the modulation of cellular iron homeostasis. Cellular iron homeostasis is regulated mainly by the import, export, and storage of iron. IREB2 is an iron regulatory protein that can be deubiquitinated by OTUD1 to upregulate TFRC, which interacts with ferric iron-loaded transferrin to promote iron uptake and ferroptosis. Conversely, USP35 functions as a ferroptosis suppressor through stabilizing the iron exporter ferroportin. Ferritin is an iron storage protein, and NCOA4 can drive the autophagic degradation of ferritin to increase the labile iron pool. Notably, USP14 has been reported to block autophagic flux, but whether USP14 inhibits ferroptosis by targeting ferritinophagy warrants further investigation. * IREB2: iron responsive element binding protein 2; NCOA4: nuclear receptor coactivator 4; OTUD1: OTU deubiquitinase 1; TFRC: transferrin receptor protein; USP14: ubiquitin-specific protease 14; USP35: ubiquitin-specific protease 35.

Iron is an important trace element involved in physiological functions, such as DNA synthesis, DNA repair, oxygen transport, and cellular respiration 33. Despite its crucial role in physiology, abnormal iron accumulation might be cytotoxic because the labile iron pool (LIP) can react with hydrogen peroxide (H_2_O_2_) to produce hydroxyl radicals (OH^-^) through the Fenton reaction, which potently facilitates lipid peroxidation 34, 35. Hence, cellular iron homeostasis is tightly controlled to prevent unwanted damage. In general, iron homeostasis can be regulated mainly by the (1) import, (2) export, and (3) storage of iron. Transferrin receptor protein 1 (TFRC) interacts with ferric iron-loaded transferrin to facilitate cellular iron uptake, and its expression is strictly regulated by iron-sensing proteins (e.g., iron responsive element binding protein 2 (IREB2)). Under a low level of intracellular iron, IREB2 can stabilize the mRNA transcript of *TFRC*; in contrast, excess intracellular iron results in the ubiquitin-mediated degradation of IREB2 by FBXL5, a component of the E3 ligase 20, 36, 37. OTU deubiquitinase 1 (OTUD1) has been reported to increase the susceptibility of colorectal cancer (CRC) to ferroptosis, enhancing antitumor immunity through the deubiquitination of IREB2 **(Figure 1)**. Moreover, OTUD1-mediated tumor regression was observed in BALB/c mice injected with CT26 cells but not in NOD-SCID mice, indicating immune-dependent tumor clearance 20.

Ferroportin (FPN) is the only iron exporter identified in mammalian cells, and FPN knockdown sensitizes neuroblastoma cells to erastin-mediated ferroptosis 38. Tang and colleagues reported that USP35 can directly stabilize FPN via its deubiquitinase activity **(Figure 1)**; conversely, USP35 knockdown improves the sensitivity of lung cancer to paclitaxel (PTX) and cisplatin 19. Intriguingly, FPN downregulation has been reported in human lung adenocarcinoma and squamous cell carcinoma 39, and it is likely that tumor cells attempt to increase iron retention and meet the demand for iron. Indeed, cancer cells are addicted to iron to maintain their aggressiveness 40, 41, but this makes them highly susceptible to ferroptosis. It seems that cancer cells strike a balance between iron homeostasis and the prevention of ferroptosis*.*

Ferritin is an iron storage protein consisting of a heavy chain and light chain, and nuclear receptor coactivator 4 (NCOA4, a selective cargo receptor) can drive the autophagic degradation of ferritin (termed “ferritinophagy”) to promote LIP and subsequent ferroptosis 42. USP14 has been reported to inhibit autophagic flux by promoting the K63-linked deubiquitination of beclin-1 43. Notably, 6-gingerol (a bioactive component of ginger) effectively suppressed lung tumor growth by downregulating USP14, which was associated with increased expression of autophagy-related and ferroptosis-related proteins 44. These findings suggest that USP14 inhibition may trigger autophagy-dependent ferroptosis, which warrants further investigation 43, 44 **(Figure 1)**. Taken together, iron accumulation is considered an Achilles heel of cancer cells, and targeting specific DUBs to interfere with iron homeostasis seems promising for improving chemotherapy efficacy.

### Section 1.02. Cysteine metabolism

Owing to the increased demand for iron to sustain malignancy 40, 41, cancer cells are predisposed to high oxidative stress. Thus, sufficient antioxidants (e.g., GSH) are essential to balance redox homeostasis; in particular, cysteine is the rate-limiting precursor of GSH synthesis. However, de novo synthesis of cysteine is insufficient to meet the cellular demand; to this end, the import of cystine (an oxidized form of cysteine) for further GSH synthesis is indispensable 45. Systemic Xc-, comprising two subunits, namely, SLC7A11 (also known as xCT) and SLC3A2, is a cystine-glutamate antiporter that facilitates the uptake of cystine 46. The inhibition of SLC7A11 suppresses tumor progression by increasing susceptibility to ferroptosis 47, 48. Recently, a growing body of research has suggested that DUBs play a role in regulating ferroptosis by targeting SLC7A11. The mechanisms by which DUBs modulate SLC7A11 are mainly through (1) direct deubiquitination, (2) indirect regulation, and (3) a noncanonical manner (i.e., DUBs regulate SLC7A11 levels in a deubiquitinase-independent manner).

**Figure 2:**
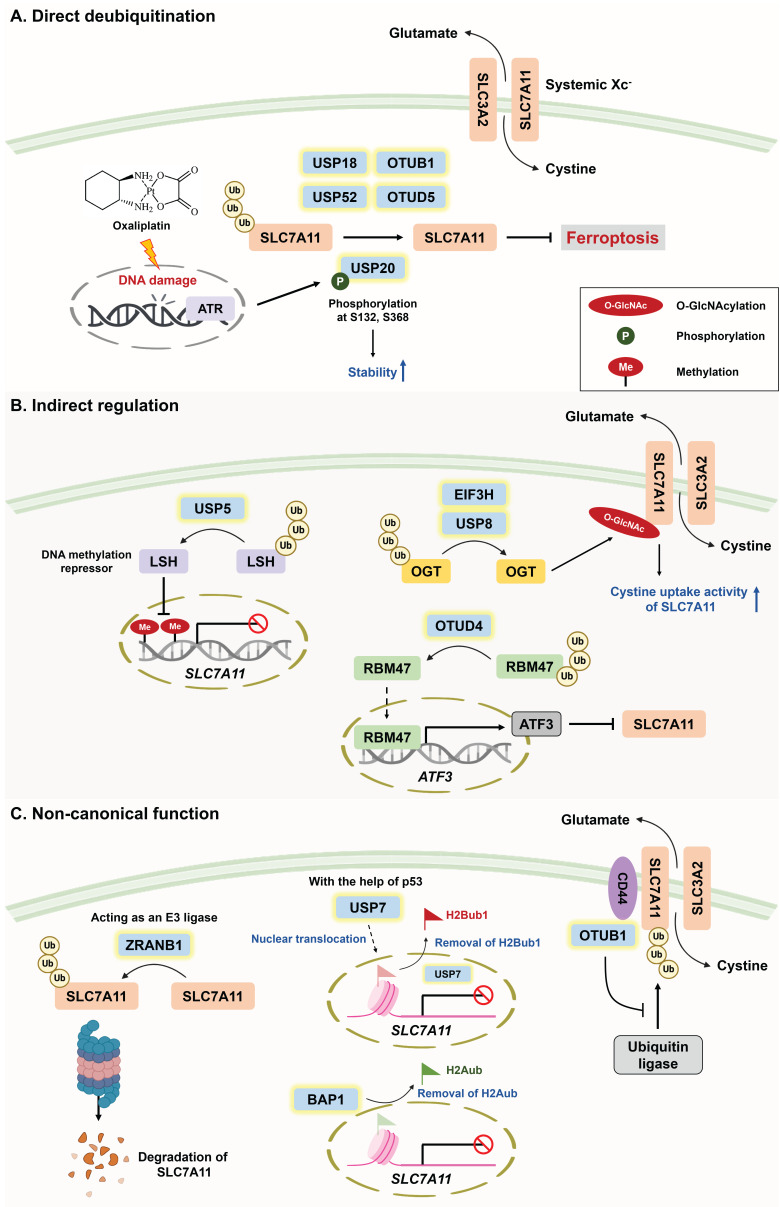
** (A)** Roles of DUBs in ferroptosis through direct deubiquitination of SLC7A11. SLC7A11 is an important subunit of systemic Xc^-^ that is responsible for cystine import and hence inhibits ferroptosis. USP18, USP20, USP52, OTUB1, and OTUD5 are capable of increasing SLC7A11 stability via their DUB activities. Notably, chemotherapeutic agent-induced ATR activation mediates USP20 phosphorylation at Ser132 and Ser368 to increase its stability, which explains why acquired oxaliplatin resistance might stem from the DNA damage-induced ATR/USP20/SLC7A11 axis. **(B)** Roles of DUBs in ferroptosis through indirect regulation of SLC7A11. In addition to being directly deubiquitinated by DUBs, SLC7A11 can be transcriptionally or posttranslationally regulated by the substrates of DUBs. LSH is both the substrate of USP5 and a DNA methylation repressor that facilitates SLC7A11 expression. OTUD4-mediated deubiquitination of RBM47 enhances the mRNA stability of ATF3, ultimately suppressing ferroptosis. In addition, USP8 and EIF3H deubiquitinate OGT for the O-GlcNAcylation of SLC7A11, increasing the cystine uptake activity of SLC7A11 and attenuating ferroptosis through reducing ROS accumulation. **(C)** Roles of DUBs in modulating SLC7A11 via their noncanonical functions. An increasing number of studies have suggested the noncanonical functions of DUBs in regulating SLC7A11, including serving as E3 ligases, epigenetic modifications, and the inhibition of ubiquitin ligases. Surprisingly, ZRANB1, which belongs to the OUT family, acts as an E3 ligase to destabilize SLC7A11 and hence increases susceptibility to ferroptosis. With respect to epigenetic modifications, with the help of p53, the nuclear translocation of USP7 removes H2Bub1 and downregulates SLC7A11. Moreover, BAP1 is a well-recognized DUB of histone H2A and is involved in SLC7A11 repression. Intriguingly, CD44 promotes the interaction between OTUB1 and SLC7A11, which stabilizes SLC7A11 by inhibiting ubiquitin ligases independent of OTUB1 DUB activity. *A20: TNF-α-induced protein 3; ATF3: activating transcription factor 3; ATR: ataxia telangiectasia and Rad3-related protein; BAP1: BRCA1-associated protein 1; CD44: cluster of differentiation 44; DUBA: deubiquitinating enzyme A; EIF3H: eukaryotic translation initiation factor 3, subunit H; LSH: lymphoid-specific helicase; OGT: O-linked N-acetylglucosamine transferase; OTUB1: OTU deubiquitinase, ubiquitin aldehyde binding 1; OTUD4: OTU deubiquitinase 4; OTUD5: OTU deubiquitinase 5; RBM47: RNA binding motif protein 47; SENP1: SUMO-specific peptidase 1; USP5: ubiquitin-specific protease 5; USP8: ubiquitin-specific protease 8; USP20: ubiquitin-specific protease 20; USP52: ubiquitin-specific protease 52; ZRANB1: zinc finger RANBP2-type containing 1.

With respect to the direct deubiquitination of SLC7A11 **(Figure 2A)**, USP18 has been reported to maintain SLC7A11 stability by counteracting CRL3^KCTD10^ E3 ligase-mediated ubiquitylation 49. As mentioned above, susceptibility to ferroptosis determines treatment sensitivity. OTU deubiquitinase 5 (OTUD5) and USP52 have been shown to stabilize SLC7A11 by removing its K48-linked polyubiquitination; however, the depletion of *OTUD5* and *USP52* sensitizes TNBC and bladder cancer to PTX and imidazole ketone erastin (IKE), respectively 50, 51. Compared with erastin, IKE is a potent ferroptosis inducer because of its superior solubility and metabolic stability in vivo 52, 53. Cancer stem cells (CSCs) drive tumor initiation, drug resistance, metastasis, and the immunosuppressive TME 54. To maintain their self-renewal ability, CSCs become more addicted to iron, but this increases susceptibility to ferroptosis. To this end, CSCs exploit DUBA (also known as OTUD5) to stabilize SLC7A11 via its deubiquitinase activity, which not only prevents ferroptosis but also promotes stem cell-like properties through c-Myc signaling 55. It seems promising that DUBA inhibition can synergize with ferroptosis inducers (e.g., sorafenib) to attenuate hepatocellular carcinoma (HCC) stemness.

PTMs, such as phosphorylation, SUMOylation, methylation, and ubiquitination, are responsible for protein diversity by altering protein activity, stability, subcellular localization, and interactions with binding partners 56. Interestingly, recent studies have suggested that PTMs of DUBs (e.g., phosphorylation and SUMOylation) can impact their stability, activity, and even interaction with SLC7A11, which further regulates cellular susceptibility to ferroptosis 57, 58. For example, the phosphorylation of USP20 at Ser132 and Ser368 by ataxia telangiectasia and Rad3-related (ATR) activation enhances its stability and then confers resistance to ferroptosis via the deubiquitination of SLC7A11. This finding implies that acquired oxaliplatin (OXA) resistance in HCC might result from the DNA damage-induced ATR/USP20/SLC7A11 axis 57. Indeed, the DNA repair machinery is responsible for OXA resistance 59. SUMOylation refers to the conjugation of small ubiquitin-like modifiers (SUMOs) to target proteins. Notably, SUMO-specific peptidase 1 (SENP1) is a well-documented protease that specifically deconjugates SUMO from SUMOylated targets, and Gao et al. reported a novel network between the SUMOylation of DUBs and ferroptosis regulation. Mechanistically, SENP1-mediated deSUMOylation of A20 potentially affects its interaction with SLC7A11, ultimately inhibiting ferroptosis 58. However, the detailed mechanism by which SLC7A11 levels are regulated by A20 requires further exploration.

With respect to the indirect regulation of SLC7A11 by DUBs **(Figure 2B)**, USP5 has been shown to stabilize lymphoid-specific helicase (LSH, a DNA methylation repressor), which transcriptionally upregulates SLC7A11 to inhibit ferroptosis in HCC 60. Conversely, activating transcription factor 3 (ATF3) has been identified as a ferroptosis inducer that mitigates chemoresistance because of its capacity to repress *SLC7A11* expression by binding with its promoter 61, 62. Li and colleagues reported that OTU deubiquitinase 4 (OTUD4) promotes ATF3-dependent ferroptosis and suppresses ccRCC metastasis; however, whether SLC7A11 is involved in OTUD4-mediated ferroptosis has not been determined 63. In addition to transcriptional regulation, the PTM of SLC7A11 has also been suggested to determine cellular susceptibility to ferroptosis. O-GlcNAcylation is a reversible PTM that is catalyzed by O-GlcNAc transferase (OGT) to affect the serine or threonine residues of target proteins 64. Intriguingly, O-GlcNAcylation seemingly plays a dual role in ferroptosis induction depending on its target 65-67. On the one hand, O-GlcNAcylation prevents the degradation of yes-associated proteins, which upregulate TFRC expression to increase susceptibility to ferroptosis 67. On the other hand, USP8 and EIF3H (belonging to the JAMM subfamily) have been found to suppress ferroptosis via the deubiquitination of OGT 68, 69. Mechanistically, the USP8-mediated O-GlcNAcylation of SLC7A11 enhances its ability to take up cystine, thus inhibiting ferroptosis. Conversely, genetic knockout or pharmacological inhibition of USP8 not only triggers ferroptosis but also inhibits the invasion and stemness of HCC 67. Furthermore, elevated OGT expression is strongly associated with HCC progression 70.

Recent findings have revealed the novel regulation of SLC7A11 by DUBs via an epigenetic mechanism **(Figure 2C)**. Histone H2B monoubiquitination (H2Bub1) is an important epigenetic modification that is widely recognized as a transcriptional activator 71. Under ferroptotic stress, p53 facilitates the nuclear translocation of USP7, which reduces the level of H2Bub1 in the SLC7A11 gene regulatory region and downregulates SLC7A11 expression 23. Furthermore, BRCA1-associated protein 1 (BAP1), a member of the UCH subfamily, is a well-known DUB of histone H2A, and this deubiquitination results in the repression of SLC7A11 72.

In addition to epigenetic regulation, DUBs are reported to govern SLC7A11 expression independent of their deubiquitinase activities** (Figure 2C)**. Liu and associates reported that inactivation of the catalytic activity of OTUB1 (C91A) does not decrease SLC7A11 levels, whereas disrupting the interaction between the E2-conjugating enzyme and OTUB1 (D88A) significantly reduces the stability of SLC7A11 73. These findings suggest that OTUB1 might stabilize SLC7A11 by directly targeting ubiquitin ligases instead of by its enzymatic activity, an effect that is in line with previous studies revealing that OTUB1 blocks the ubiquitin-conjugating activity of E2 74, 75. In addition, the PTM of OTUB1 affects its binding affinity with the E2 enzyme. For example, the methylation of OTUB1 compromises its binding with UBC13 (an E2-conjugating enzyme), eventually abolishing the suppressive role of OTUB1 in ferroptosis. However, the authors did not clarify which ferroptosis-related regulator is targeted by OTUB1 76. Strikingly, ZARNB1 (a member of the OTU subfamily) can function as an E3 ligase to negatively regulate SLC7A11, and its overexpression markedly enhances susceptibility to ferroptosis 77. Notably, A20 is the only member of the OTU family that possesses both DUB and E3 ligase activities 78, and the author revealed that the specific region (residues 463-584) within the OTU domain of ZARNB1 functions as an atypical E3 ligase 77.

### Section 1.03. Calcium homeostasis

Emerging studies have demonstrated that elevated levels of cytosolic Ca^2+^ increase cellular susceptibility to ferroptosis by facilitating the membrane integration of 12/15-lipoxygenase into the mitochondria and endoplasmic reticulum and that the inhibition of Ca^2+^ influx by cobalt chloride prevents erastin-induced ferroptosis 79, 80. These findings suggest the involvement of calcium homeostasis in ferroptosis regulation. A recent finding indicated that the USP11-mediated deubiquitination of LSH reduces H3K27me3 levels on the *CYP24A1* promoter, which blocks cytosolic Ca^2+^ influx and ferroptosis initiation in CRC 81. However, glucose starvation downregulates the USP11/LSH/CYP24A1 signaling axis to promote ferroptosis. Mitoxantrone, an FDA-approved antineoplastic agent, is a potent USP11 inhibitor that eliminates pancreatic ductal adenocarcinoma cells. Hence, in combination with the manipulation of glucose metabolism, USP11 might be a promising druggable target for CRC treatment 82, 83.

### Section 1.04. Lipid metabolism

**Figure 3:**
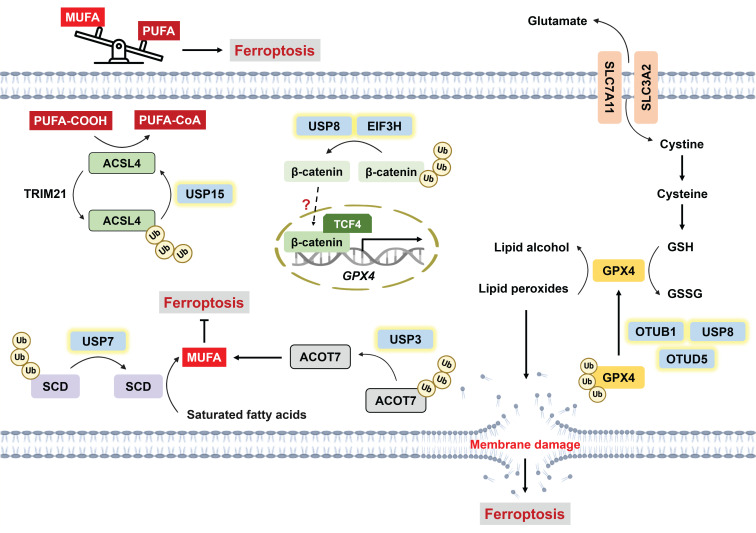
Roles of DUBs in ferroptosis through the modulation of lipid metabolism. The balance between MUFAs and PUFAs within phospholipids determines the cellular susceptibility to ferroptosis, and PUFA enrichment induces lipid peroxidation and ferroptosis. On the one hand, USP15 counteracts TRIM21-mediated polyubiquitination of ACSL4 to increase its stability, which facilitates the incorporation of PUFA-CoAs into the plasma membrane. On the other hand, USP7 and USP3 increase MUFA production by deubiquitinating SCD and ACOT7, respectively. GPX4 is a well-recognized ferroptosis suppressor that converts lipid peroxides into lipid alcohol by oxidizing GSH to generate GSSG. USP8, OTUB1 and OTUD5 can deubiquitinate and stabilize GPX4. In addition, USP8 and EIF3H were reported to confer ferroptosis resistance via the deubiquitination of β-catenin, but the precise mechanism remains unclear. USP8 likely negatively regulates ferroptosis via the USP8/β-catenin/GPX4 axis. *ACOT7: acyl-CoA thioesterase 7; ACSL4: long-chain acyl-CoA synthetase-4; GSH: glutathione; GSSG: glutathione disulfide; MUFAs: monounsaturated fatty acids; PUFAs: polyunsaturated fatty acids; SCD: stearoyl-CoA desaturase; TCF4: transcription factor 4; TRIM21: tripartite motif-containing protein 21; USP3: ubiquitin-specific protease 3; USP7: ubiquitin-specific protease 7; USP15: ubiquitin-specific protease 15.

As mentioned above, intracellular labile iron can promote the generation of hydroxyl radicals via the Fenton reaction, thereby inducing lipid peroxidation 34, 35. Compared with monounsaturated fatty acids (MUFAs), polyunsaturated fatty acids (PUFAs) are more susceptible to peroxidation. That is, increased levels of less oxidizable MUFAs result in ferroptosis resistance 84. The regulation of ferroptotic cell death through lipid metabolism has been fully elucidated previously 85. In brief, the balance between MUFAs and PUFAs within phospholipids determines the sensitivity of ferroptosis, which can be modulated by lipid metabolic enzymes. Stearoyl-CoA desaturase (SCD) catalyzes fatty acid desaturation to facilitate MUFA accumulation, and SCD overexpression has been reported to prevent ferroptosis 86-89. Guan et al. revealed that USP7 is capable of deubiquitinating SCD; conversely, the inhibition of SCD by DHPO (an allosteric site covalent inhibitor of USP7) not only induces ferroptosis but also exhibits superior antitumor efficacy without severe side effects compared with cisplatin in a gastric cancer PDX mouse model 24. Furthermore, USP3 has been found to suppress ferroptosis and enhance NSCLC resistance to cisplatin via the deubiquitination of acyl-CoA thioesterase 7 (ACOT7), a MUFA synthesis-related enzyme 90 **(Figure 3)**.

Long-chain acyl-CoA synthetase (ACSL) is an enzyme responsible for remodeling phospholipid composition through the conversion of free fatty acids into fatty acyl-CoAs. In particular, ACSL4 preferentially activates PUFAs, which facilitates their incorporation into the plasma membrane 91. The inhibition of ACSL4 has been reported to suppress lipid peroxidation as well as ferroptosis 92, 93; hence, increased enzymatic activity or cellular levels of ACSL4 fuel the proferroptotic process. ACSL4 stability is clearly governed by the balance between the E3 ligases TRIM21 and USP15. Mechanistically, TRIM21 catalyzes, but USP15 removes, the K48-linked polyubiquitination of ACSL4, resulting in ACSL4 degradation and stabilization, respectively 94 **(Figure 3)**. Tyrosine-protein kinase KIT mutations are predominantly observed in 85% of gastrointestinal stromal tumors (GISTs) 95. Notably, c-KIT activation induces the upregulation of TRIM21 expression but the downregulation of USP15 expression, which destabilizes ACSL4 and is associated with the resistance of GIST to imatinib (a c-KIT inhibitor) 94.

Glutathione hydroperoxidase 4 (GPX4), a negative regulator of ferroptosis, is capable of converting lipid peroxides into lipid alcohols by oxidizing GSH to glutathione disulfide (GSSG) 96. An increasing number of studies have suggested the involvement of DUBs in regulating GPX4-dependent ferroptosis **(Figure 3)**. For example, USP8 stabilizes GPX4 via its enzymatic activity. The combination of DUB-IN-2 (a USP8 inhibitor) and sulfasalazine (SAS, a ferroptosis inducer) sensitizes cells to PD-1 inhibitors and increases IFN-γ^+^ CD8^+^ T-cell infiltration 97. Another study revealed that the broad-spectrum DUB inhibitor (DUBi) PR-619 induces GPX4 destabilization, which promotes tumor-infiltrating CD8^+^ T cells and potentiates immunotherapy 98. These findings highlight the potential of ferroptosis to augment antitumor immunity. OTUD5 is reportedly involved in ferroptosis-mediated renal and myocardial ischemia/reperfusion injury 99, 100. A recent finding suggests that p53-mediated transcriptional inhibition of OTUD5 destabilizes GPX4 and confers susceptibility to ferroptosis in stomach adenocarcinoma 101. USP2, which is usually suppressed in cisplatin-resistant NSCLC, can directly interact with p53 to promote its stability and nuclear translocation, eventually facilitating ferroptosis 102. p53 functions as a bidirectional regulator of ferroptosis depending on cellular stress. On the one hand, p53 downregulates SLC7A11 and indirectly represses GPX4 activity via reduced GSH synthesis; on the other hand, it can increase GPX4 activity via p21-induced GSH production to maintain cell survival 103. Several E3 ligases have been implicated in regulating GPX4 stability through alterations in its ubiquitination status 104, 105. For example, the interaction between tripartite motif-containing 41 (TRIM41) and GPX4 facilitates GPX4 degradation 104. Conversely, tripartite motif-containing 26 (TRIM26) enhances GPX4 stability by catalyzing K63-linked ubiquitination 105. However, the interplay between DUBs and E3 ligases and their cooperative mechanisms in orchestrating ubiquitin networks to regulate cell death require further investigation.

The cystatin SN is positively correlated with enhanced metastasis in GC, and it serves as a ferroptosis suppressor through the recruitment of OTUB1 to deubiquitinate GPX4 106. USP8 and EIF3H have also been found to confer ferroptosis resistance by deubiquitinating β-catenin, which is associated with cancer stemness and invasion 107, 108. In addition, Wang et al. demonstrated that β-catenin directly targets the promoter of GPX4, thereby upregulating GPX4 expression and subsequently inhibiting lipid ROS generation 109. Therefore, ferroptosis may be negatively regulated by the USP8/β-catenin/GPX4 axis, but this hypothesis warrants further testing. Similarly, USP15-mediated deubiquitination of selenium-binding protein 1 (SELENBP1) contributes to ferroptosis resistance, but the underlying mechanism remains elusive 110. USP15 may modulate ferroptosis via the upregulation of GPX4 expression via SELENBP1 111.

The ferroptosis suppressor protein 1 (FSP1)-coenzyme Q10 (CoQ10) system is also a negative regulator of lipid peroxidation independent of the GSH-GPX4 pathway. Mechanistically, FSP1 inhibits lipid hydroperoxides by converting CoQ10 (ubiquinone) into its reduced form CoQ10H_2_ (ubiquinol), which scavenges free radicals 112. Blockade of FSP1 not only triggers ferroptotic cell death but also enhances the efficacy of ICB 113. A recent study demonstrated that USP7 deubiquitinates JunD, which transcriptionally upregulates FSP1 and confers ferroptosis resistance in melanoma 114. However, whether any DUB directly stabilizes FSP1 through its deubiquitination warrants further investigation.

### Section 1.05. Nrf2 signaling pathway

Nrf2 is an important transcription factor that regulates cellular redox homeostasis, and its stability is tightly controlled by the KEAP1-CUL3-RBX1 E3 ligase complex. Following nuclear translocation, Nrf2 binds with small Mafs to form a heterodimer and then targets the antioxidant response element, which increases the expression of downstream antioxidant genes to counteract oxidative stress 115. The key negative regulators of ferroptosis, including SLC7A11 and GPX4, are Nrf2 target genes 116, 117; furthermore, Nrf2 has been found to inhibit ferroptosis by promoting SLC7A11 membrane localization in an autophagy-dependent manner 117, 118. These findings highlight the suppressive role of Nrf2 signaling in ferroptosis. Recently, several DUBs, including USP11, USP35, and OTUD3, have been shown to reduce cellular susceptibility to ferroptosis by deubiquitinating Nrf2 14, 119, 120.

Overall, DUBs play dual roles in regulating ferroptosis depending on their target proteins or involved mechanisms, and an increasing number of studies have reported the noncanonical functions of DUBs in ferroptosis modulation. Thus, an in-depth understanding of the crosstalk between DUBs and ferroptosis will provide insight into cancer therapeutics.

## Article II. The role of DUBs in the modulation of pyroptosis

**Figure 4:**
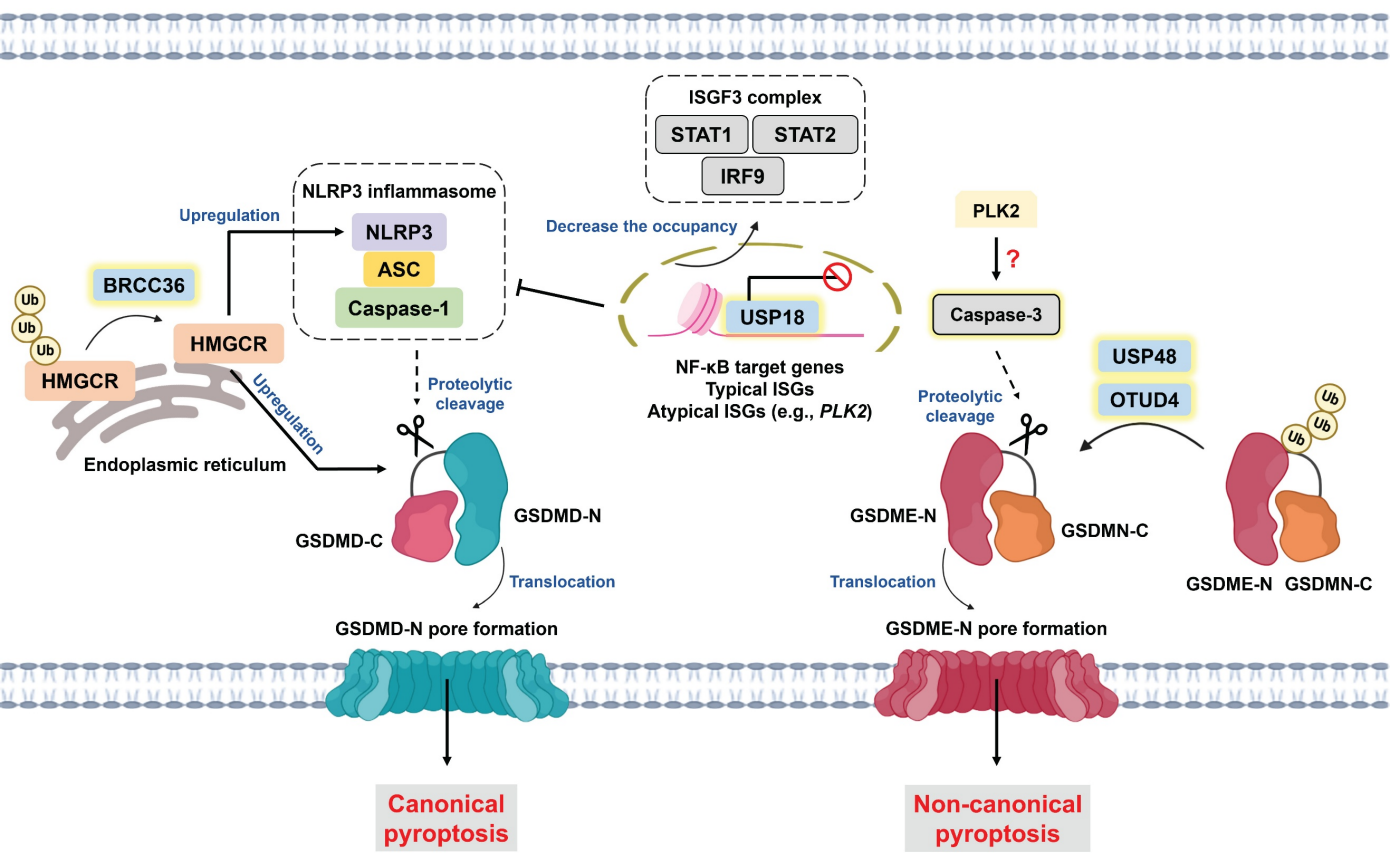
Roles of DUBs in pyroptosis modulation. Pyroptosis can be classified into canonical and noncanonical signaling pathways on the basis of its molecular mechanism. Canonical pyroptosis is initiated by an inflammasome consisting of PRR, ASC, and caspase-1. Active caspase-1 cleaves GSDMD to liberate GSDMD-N, contributing to pore formation on the plasma membrane. For noncanonical pyroptosis (the LPS/caspase-4/-5/GSDMD axis is not shown here), active caspase-3 induces the cleavage of GSDME to facilitate GSDME-N generation and subsequent pore formation. Notably, the cellular level of GSDME is the determining factor in the cell death switch between apoptosis and pyroptosis. USP48 and OTUD4 deubiquitinate GSDME to increase its level and promote pyroptosis. BRCC36 serves as a key player in controlling the switch between ferroptosis and pyroptosis. BRCC36 deubiquitinates HMGCR to trigger pyroptosis but inhibits ferroptosis via the upregulation of NLRP3 and GSDMD. Interestingly, USP18 regulates both canonical and noncanonical pyroptosis via epigenetic modifications. Nuclear USP18 prevents the ISGF3 complex (STAT1-STAT2-IRF9)-induced transcription of NF-κB target genes and typical as well as atypical ISGs (e.g., *PLK2*), which suppresses both GSDMD- and GSDME-mediated pyroptosis. *ASC: apoptosis-associated speck-like protein containing a CARD domain; BRCC36: BRCA1/BRCA2-containing complex subunit 36; GSDMD: gasdermin D; GSDMD-N: GSDMD N-terminal; GSDME: gasdermin E; GSDME-N: GSDME N-terminal; HMGCR: 3-hydroxy-3-methylglutaryl-coenzyme A reductase; IRF9: interferon regulatory factor 9; ISGF3: interferon-stimulated gene factor 3; LPS: lipopolysaccharide; NLRP3: NOD-like receptor protein 3; PLK2: Polo-like kinase 2; PRR: pattern recognition receptor; STAT1: signal transducer and activator of transcription 1; STAT2: signal transducer and activator of transcription 2; USP48: ubiquitin-specific protease 48.

Pyroptosis is a type of gasdermin (GSDM)-driven ICD characterized by the release of proinflammatory stimuli, such as interleukin-1β (IL-1β), interleukin-18 (IL-18), and damage-associated molecular patterns (DAMPs) 121. In a protein family consisting of six paralogous genes in humans, GSDMs have two distinct domains, including a pore-forming N-terminus and an autoinhibitory C-terminus. Once cleaved by specific caspases, the N-terminus of GSDMs is liberated and translocates to the plasma membrane to induce pore formation 122. Morphologically, pyroptosis is characterized by GSDM pore formation, bubble-like protrusions, cell flattening and ultimately plasma membrane rupture 123. With respect to molecular mechanisms, pyroptosis can be classified into canonical and noncanonical pathways. Canonical pyroptosis is initiated by inflammasome activation, which involves pattern recognition receptors (PRRs), apoptosis-associated speck-like protein containing a CARD (ASC), and caspase-1. Active caspase-1 not only facilitates the cleavage of gasdermin D (GSDMD) but also induces the maturation of IL-1β and IL-18 124. Noncanonical pyroptosis is triggered by the LPS-mediated activation of caspase-4/-5, which subsequently cleaves GSDMD 125. Notably, caspase-3, widely recognized as the executor of apoptosis, is capable of inducing pyroptotic cell death. In fact, several conventional chemotherapeutic agents (e.g., cisplatin, lobaplatin, and 5-FU) have been suggested to trigger GSDME-dependent pyroptosis, which potently overcomes chemoresistance and augments antitumor immunity 8, 126-129. For example, cisplatin-induced pyroptosis promotes the secretion of IL-12 and further upregulates IFN-γ expression in T cells, thereby sensitizing small lung cancer cells to PD-L1 blockade 126. Like GSDME-mediated pyroptosis, apoptosis shares upstream signaling, and the level of GSDME determines the switch between apoptosis and pyroptosis 129. GSDME serves as a favorable prognostic factor 8, whereas its expression is frequently silenced because of promoter hypermethylation 130, 131. Interestingly, USP18 has been reported to suppress pyroptosis through epigenetic regulation independent of its deubiquitinase activity. Mechanistically, nuclear USP18 abrogates the occupancy of the STAT2 transcription complex to its relative DNA motif, which significantly downregulates canonical and atypical interferon-stimulated genes (ISGs) and NF-κB target genes. Among atypical ISGs, Polo-like kinase 2 (PLK2) functions as a potential mediator of GSDME-dependent pyroptosis **(Figure 4)**. Single-cell RNA sequencing (scRNA-seq) analysis of acute myeloid leukemia cells revealed that increased USP18 expression after chemotherapy promotes cell survival, accounting for disease relapse 132.

In addition to epigenetic modifications, DUBs are engaged in regulating GSDME-mediated pyroptosis via their deubiquitinase activity **(Figure 4)**. OTUD4 enhances the radiosensitivity of nasopharyngeal cancer (NPC) since it can deubiquitinate and stabilize GSDME to induce pyroptosis. As expected, patients with low OTUD4 expression present a poorer response to radiotherapy and shorter progression-free survival 22. Moreover, Ren and colleagues exploited the CRISPR-Cas9 system and LDH secretion to determine that USP48 is a potential inducer of pyroptosis. USP48 increases the stability but not the cleavage of GSDME by removing its K48-linked ubiquitination 21. Pyroptosis is closely related to immunomodulation of the TME 121, and increased infiltration and function of NK cells and CD8^+^ T cells have been found in GSDME-expressing mouse models 133. The loss of USP48 reduces the proportions of NK cells and CD8^+^ T cells but increases tumor-associated macrophage and regulatory T (Treg) infiltration. Notably, scRNA-seq analysis revealed that exhausted T (Tex) cells and Treg cells are T-cell subpopulations that are negatively regulated by USP48 21. Hou and colleagues reported that USP47 knockdown enhances doxorubicin-mediated pyroptosis but does not affect caspase-3 activation. These findings suggest that the substrate of USP47 might be involved in preventing GSDME cleavage; however, the detailed mechanism warrants further exploration 134.

Emerging studies have revealed the relationship between ferroptosis and pyroptosis 135, 136. For example, the byproduct of lipid peroxidation 4-HNE hinders the interaction between the NLRP3 inflammasome and NIMA-related kinase 7 (NEK7), thereby inhibiting pyroptosis 136. Intriguingly, BRCA1/BRCA2-containing complex subunit 36 (BRCC36), a member of the JAMM family, has been suggested to regulate the switch between ferroptosis and pyroptosis. BRCC36-mediated deubiquitination of 3-hydroxy-3-methylglutaryl-coenzyme A reductase (HMGCR) induces pyroptosis but inhibits ferroptosis. Notably, the subcellular localization of HMGCR determines the mode of cell death. HMGCR is predominantly localized in mitochondria; however, HMGCR is located primarily in the endoplasmic reticulum to induce pyroptosis via the upregulation of NLRP3 and GSDMD expression **(Figure 4)** 137. The administration of thiolutin (THL, a BRCC36 inhibitor synthesized from Streptomyces) 138 effectively suppresses HCC progression and augments RSL3-induced ferroptosis 137. These findings reveal the dark side of pyroptosis and indicate that intensive pyroptosis plays a tumor-suppressive role, whereas chronic pyroptosis might instead facilitate tumor progression 139.

## Article III. Discussion

Over the past few decades, most chemotherapeutics have been introduced to eliminate cancer cells by targeting the apoptosis signaling pathway 1. However, aberrant apoptotic machinery is recognized as a significant hallmark of cancer that drives therapy resistance 140. Recently, the induction of nonapoptotic PCD (e.g., ferroptosis, necroptosis, and pyroptosis) has been proven to not only effectively kill chemoresistant cells but also improve the sensitivity of tumors to clinically approved chemotherapeutic agents 6, 7, 141. Capable of facilitating the release of proinflammatory stimuli, nonapoptotic PCD potentially relieves immunosuppression and augments the efficacy of ICB 142, 143. Ubiquitination and deubiquitination are important PTMs for the orchestration of cellular homeostasis. Nevertheless, emerging evidence has revealed the dysregulation of DUBs in tumor progression 15-17 and their involvement in regulating cellular sensitivity to ferroptosis and pyroptosis 14, 20-22. In fact, susceptibility to nonapoptotic PCD, to some extent, determines the effectiveness of chemotherapy and ICB and is correlated with tumorigenesis 28, 126. Pharmacologically or genetically targeting specific DUBs has been reported to inhibit tumor progression (e.g., stemness and metastasis), augment antitumor immunity, and synergize with clinically used drugs to eliminate tumors 21, 68, 97, 134. For example, DUB-IN-2 treatment not only promotes the infiltration of cytotoxic T cells but also sensitizes multiple murine tumor models to anti-PD-1 therapy through the induction of ferroptosis 97. Moreover, USP47 knockdown enhances the chemosensitivity of CRC to doxorubicin by triggering pyroptosis 134. Notably, owing to their regulatory roles in nonapoptotic PCD, DUBs can function as prognostic indicators and potent biomarkers of drug sensitivity and the immune landscape within the TME 20-22, 57, 90. Overall, in this review, we highlight DUBs as potential regulators of ferroptosis and pyroptosis and their implications for cancer treatment.

Although targeting specific DUBs seems promising for blocking tumor progression and improving drug effectiveness through increasing susceptibility to ferroptosis and pyroptosis, several urgent problems and challenges are worth discussion and warrant further exploration. (i) In addition to reporting on ferroptosis and pyroptosis, studies have reported the involvement of DUBs in regulating apoptosis and other nonapoptotic PCDs (e.g., necroptosis and paraptosis) 144-149. For instance, USP8 inhibits extrinsic apoptosis by deubiquitinating the long isoform of FLICE-inhibitory protein (FLIP_L_) and promoting the activation of the human epidermal growth factor receptor (HER) family 150, 151. Additionally, USP8 serves as an independent prognostic indicator for adverse outcomes in patients with ovarian carcinoma 151. USP7, another negative regulator of apoptosis, deubiquitinates and stabilizes the antiapoptotic protein ADP-ribosylation factor 4 (ARF4) to inhibit BAX 152. OTUB1 has been found to suppress necroptosis by deubiquitinating β-catenin. The nuclear translocation of β-catenin downregulates the expression of key executors of necroptosis, including receptor-interacting serine-threonine kinase 3 (RIPK3) and mixed lineage kinase domain-like pseudokinase (MLKL), which facilitate cisplatin resistance 144. USP10 was identified as a positive regulator of curcumin-mediated paraptosis characterized by mitochondrial dilation, but the molecular targets of USP10 remain unclear 148. Therefore, the roles of DUBs in modulating other types of PCD and their implications for cancer treatment should not be overlooked. Notably, the influence of DUBs on cell fate, specifically different types of cell death, is also worth discussing. One salient example is receptor-interacting serine-threonine kinase 1 (RIPK1), whose ubiquitination status to some extent determines cell fate 153. Deubiquitination of RIPK1 by the tumor suppressor DUB cylindromatosis (CYLD) can facilitate apoptosis or necroptosis, whereas A20 instead promotes RIPK1 degradation, thereby sustaining cell survival 154. These findings suggest that the precise activity of DUBs and diverse cellular contexts influence survival, apoptosis, and necroptosis in response to TNF-α signaling. Upon caspase-3 activation, the level of GSDME is involved in the cell death switch between apoptosis and pyroptosis. In other words, cells with high levels of GSDME preferentially undergo pyroptosis 155. As mentioned above, USP48 and OTUD4 deubiquitinate GSDME to ensure its stability 21, 22; hence, the cellular levels of USP48 and OTUD4 determine the fate of cells in terms of apoptosis or pyroptosis. Moreover, DUBs might influence cell fate by modulating the threshold to induce cell death. For example, the delicate balance between proapoptotic and antiapoptotic proteins regulates the initiation of apoptosis 156. If the substrate of a DUB (e.g., USP27x) is a proapoptotic protein (e.g., Bim), the abundance of this DUB might facilitate apoptosis and improve the treatment response, and vice versa 149.

(ii) The interplay between DUBs and their substrates is complicated. On the one hand, numerous DUBs are capable of targeting identical proteins. For example, SLC7A11 can be stabilized by DUBA and OTUB1 to confer ferroptosis resistance 55, 73. Additionally, β-catenin is deubiquitinated by USP8 and OTUB1 to inhibit ferroptosis and necroptosis, respectively 107, 144. Notably, USP2a, USP20, and USP47 have also been reported to deubiquitinate and stabilize β-catenin 157-159, and it is questionable whether these DUBs can similarly regulate ferroptosis or necroptosis in a β-catenin-dependent manner. On the other hand, a DUB can target diverse proteins or activate distinct signaling pathways on the basis of the cellular context. One salient example is OTUD4. Li et al. demonstrated that OTUD4 promotes ferroptosis via the RBM47/ATF3/SLC7A11 axis in ccRCC 63, whereas OTUD4 triggers pyroptosis through stabilizing GSDME in NPC 22. Furthermore, USP7 plays dual roles in regulating ferroptosis, likely through different targets and mechanisms. With the help of p53, the nuclear translocation of USP7 induces ferroptosis by epigenetically repressing SLC7A11 expression 23; in contrast, USP7 blocks ferroptosis by deubiquitinating SCD and thus inhibiting lipid ROS 24. With respect to the challenges of targeting DUBs, most DUBs share similar catalytic pockets 160, and emerging evidence has revealed the noncanonical functions of DUBs without presenting catalytic pockets 76, 161. Owing to the abovementioned reasons, it is difficult to find highly selective DUB inhibitors, which explains why most DUB inhibitors are multitargeted and are likely to cause intolerable side effects 162. Nevertheless, recent advances in DUBi development have been reported. Chan and colleagues created a covalent library with chemical diversification to target multiple and discrete areas around the catalytic sites of DUBs. Combined with activity-based protein profiling (ABPP), this approach accelerated DUBi development, leading to the identification of a covalent inhibitor of the understudied DUB VIPCP1 with nanomolar potency 163. Moreover, proteolysis-targeting chimeras (PROTACs) are heterobifunctional molecules consisting of two distinct ligands that bridge the target protein and an E3 ligase in close proximity, leading to degradation of the target protein. Thus, some undruggable DUBs might be eliminated by PROTACs 164. Notably, the PROTAC U7D-1 was shown to selectively degrade USP7, effectively inhibiting cancer cell growth 165.

(iii) Although the induction of ferroptosis and pyroptosis significantly inhibits tumor progression, the dark side of targeting these nonapoptotic modes of PCD has been reported, possibly on the basis of the context of the TME or types of released DAMPs. For example, ferroptotic pancreatic ductal adenocarcinoma (PDAC) cells release exosomal KRAS oncoproteins, subsequently inducing macrophage M2 polarization 166. High mobility group box 1 (HMGB1) derived from pyroptotic cells facilitates the tumorigenesis of colitis-associated CRC 167. Neutralization of protumorigenic DAMPs seems to be a solution to this. For example, 8-hydroxyguanosine (8-OHG) released from ferroptotic PDAC cells can facilitate increased infiltration of M2 macrophages, whereas the administration of anti-8-OHG antibodies inhibits ferroptosis-mediated tumorigenesis in *Kras*-driven mice 168. Accurately targeting DUBs to induce nonapoptotic PCD within tumor cells is also a major concern since the simultaneous nonselective induction of ferroptosis in cancer cells and immune cells causes immunosuppression 96, 169, 170. RSL3, a GPX4 inhibitor, might trigger ferroptosis in dendritic cells, which results in reduced expression of proinflammatory cytokines and MHC I and limited functions of CD8^+^ T cells 171. The selective elimination of cancer cells by the induction of ferroptosis or pyroptosis could be achieved via the use of antibody‒drug conjugates or nanoparticle-based delivery systems, which target proteins that are preferentially expressed in cancer cells 172, 173. For example, a nanogel containing the ferroptosis inducer IKE conjugated to an anti-PD-L1 antibody was shown to trigger ferroptosis in PD-L1-expressing cancer cells, resulting in greater specificity than systemic delivery of IKE 172. Furthermore, N6F11 was identified as a selective ferroptosis inducer that specifically degrades GPX4 via TRIM25-mediated ubiquitination in cancer cells because of its preferential expression 96.

Notably, xenograft mouse models bearing human tumors are often used to evaluate the antitumor effects of nonapoptotic PCD. However, the negative effects of ferroptosis or pyroptosis induction by targeting DUBs might be overlooked due to the use of immunodeficient mice. Hence, the utilization of immunocompetent mouse models, including syngeneic mouse models, genetically engineered mouse models, and humanized mice with patient-derived xenografts, enables a comprehensive exploration of the nonapoptotic PCD-elicited effects on the TME 168, 174, 175.

(iv) Emerging evidence has revealed the unconventional roles of DUBs in regulating nonapoptotic PCD, such as disrupting the interaction between the E2-conjugating enzyme and SLC7A11 73, functioning as an E3 ligase 77, and being involved in epigenetic modifications 23, 132. Moreover, PTMs (e.g., phosphorylation, SUMOylation, and methylation) of DUBs potentially influence their stability, activity, or physical interaction with their binding partners 57, 58, 68, 76. Taken together, an in-depth understanding of the relationships between DUBs and the mechanisms involved in the modulation of nonapoptotic PCD will be conducive to advancing cancer treatment.

## Figures and Tables

**Table 1:** Potential small molecules that target DUBs to induce ferroptosis and inhibit tumor progression

DUB subfamily	DUB	Inhibitor/ Compound	Cancer type	The substrate of DUB	Mechanism/Effect/Implication	Ref.
JAMMs	BRCC36	Thiolutin	HCC	HMGCR	1. Inhibit BRCC36-mediated deubiquitination of HMGCR by targeting the BRCC36's enzymatic activity 2. Effectively disrupt the physical interaction between BRCC36 and HMGCR 3. Trigger ferroptosis instead of pyroptosis and inhibit tumor growth *in vivo*	137
USPs	USP5	Degrasyn	HCC	LSH	1. Inhibit USP5-mediated deubiquitination of LSH and downregulate ferroptosis-related markers (e.g., SLC7A11 and GPX4) 2. Inhibit tumor growth in nude mice subcutaneously injected with LH3/Hep3B/HepG2	60
	USP7	DHPO (2α,6α-diacetoxy-4β-hydroxy11-pseudoguaien-12,8α-olide)	GC	SCD	1. DHPO is an allosteric site covalent inhibitor of USP7 2. Inhibit USP7-mediated deubiquitination of SCD and induce ferroptosis through reducing MUFA production 3. Suppress metastasis in orthotopic mouse models bearing MGC803-Luc or MKN-1-Luc cells with no significant toxicity 4. Prolong overall survival in GC patient-derived xenograft mouse models compared to cisplatin treatment	24
	USP8	DUB-IN-2	CRC	GPX4	1. Reduce the protein level of GPX4 by inhibiting its deubiquitination 2. Combine IKE or SAS to inhibit tumor growth and increase tumoral 4-HNE expression of CT26 subcutaneous tumors 3. The number of tumor-infiltrating IFNγ^+^ or TNF^+^ CD8^+^ T cells markedly increases under triple therapy (DUB-IN-2+SAS+PD-1 mAb) in BALB/c mice bearing subcutaneous CT26 tumors	97
		DUB-IN-3	HCC	β-catenin	1. Reduce β-catenin expression and induce ferroptosis 2. Inhibit the proliferation, invasion, and stem-like properties of HCC cells	107
				OGT	1. Inhibit USP8-mediated deubiquitination of OGT and further reduce cystine uptake activity by decreasing GlcNAcylation of SLC7A11 2. Reduce intracellular cystine levels but promote ROS accumulation to trigger ferroptosis 3. Inhibit the proliferation, invasion, metastasis, and stemness of HCC cells	68
	USP11	Mitoxantrone (MTX)	CRC	LSH	Inhibit USP11-mediated deubiquitination of LSH and reduce its protein stability	81
	USP14	6-Gingerol (naturally occurring phenol in ginger)	NSCLC	N/A	1. Downregulate USP14 expression and inhibit the deubiquitination of beclin1 at the K63 site 2. Increase iron levels but reduce GPX4 protein levels	44
	USP20	GSK2643943A	HCC	SLC7A11	1. Increase the cellular sensitivity to OXA 2. Synergize with OXA to retard tumor growth in BALB/c nude mice bearing Huh-7 cells	57
--	A broad-range and reversible DUB inhibitor	PR-619	CRC	N/A	1. Promote GPX4 degradation and induce ferroptosis 2. Combine anti PD-1 mAb to facilitate the release of DAMPs and increase the infiltration of IFN-γ^+^ and granzyme B^+^ CD8^+^ T cells.	98

## Abbreviations

CRC: Colorectal cancer
DAMPs: Damage-associated molecular patterns
GC: Gastric cancer
HCC: Hepatocellular carcinoma
HMGCR: 3-Hydroxy-3-methylglutaryl-CoA reductase
IKE: Imidazole ketone erastin
LSH: Lymphoid-specific helicase
MUFA: Monounsaturated fatty acid
NSCLC: Non-small cell lung cancer
OGT: O-GlcNAc transferase
OXA: Oxaliplatin
SAS: Sulfasalazine
SCD: Stearoyl-CoA desaturase

## References

[1] Carneiro BA, El-Deiry WS (2020). Targeting apoptosis in cancer therapy. Nat Rev Clin Oncol.

[2] Holohan C, Van Schaeybroeck S, Longley DB, Johnston PG (2013). Cancer drug resistance: an evolving paradigm. Nat Rev Cancer.

[3] Chang Z, Zhang Y, Liu J, Guan C, Gu X, Yang Z (2019). GATA1 Promotes Gemcitabine Resistance in Pancreatic Cancer through Antiapoptotic Pathway. J Oncol.

[4] Williams J, Lucas PC, Griffith KA, Choi M, Fogoros S, Hu YY (2005). Expression of Bcl-xL in ovarian carcinoma is associated with chemoresistance and recurrent disease. Gynecol Oncol.

[5] Abizanda-Campo S, Virumbrales-Munoz M, Humayun M, Marmol I, Beebe DJ, Ochoa I (2023). Microphysiological systems for solid tumor immunotherapy: opportunities and challenges. Microsyst Nanoeng.

[6] Liu K, Huang J, Liu J, Klionsky DJ, Kang R, Tang D (2022). Induction of autophagy-dependent ferroptosis to eliminate drug-tolerant human retinoblastoma cells. Cell Death Dis.

[7] Guo J, Zheng J, Mu M, Chen Z, Xu Z, Zhao C (2021). GW4064 enhances the chemosensitivity of colorectal cancer to oxaliplatin by inducing pyroptosis. Biochem Biophys Res Commun.

[8] Peng Z, Wang P, Song W, Yao Q, Li Y, Liu L (2020). GSDME enhances Cisplatin sensitivity to regress non-small cell lung carcinoma by mediating pyroptosis to trigger antitumor immunocyte infiltration. Signal Transduct Target Ther.

[9] Damgaard RB (2021). The ubiquitin system: from cell signalling to disease biology and new therapeutic opportunities. Cell Death Differ.

[10] Ebner P, Versteeg GA, Ikeda F (2017). Ubiquitin enzymes in the regulation of immune responses. Crit Rev Biochem Mol Biol.

[11] Komander D, Clague MJ, Urbe S (2009). Breaking the chains: structure and function of the deubiquitinases. Nat Rev Mol Cell Biol.

[12] Dewson G, Eichhorn PJA, Komander D (2023). Deubiquitinases in cancer. Nat Rev Cancer.

[13] Georges A, Gros P, Fodil N (2021). USP15: a review of its implication in immune and inflammatory processes and tumor progression. Genes Immun.

[14] Meng C, Zhan J, Chen D, Shao G, Zhang H, Gu W (2021). The deubiquitinase USP11 regulates cell proliferation and ferroptotic cell death via stabilization of NRF2 USP11 deubiquitinates and stabilizes NRF2. Oncogene.

[15] Zhu X, Zhang Y, Luo Q, Wu X, Huang F, Shu T (2021). The deubiquitinase USP11 promotes ovarian cancer chemoresistance by stabilizing BIP. Signal Transduct Target Ther.

[16] Li W, Shen M, Jiang YZ, Zhang R, Zheng H, Wei Y (2020). Deubiquitinase USP20 promotes breast cancer metastasis by stabilizing SNAI2. Genes Dev.

[17] Zhu D, Xu R, Huang X, Tang Z, Tian Y, Zhang J (2021). Deubiquitinating enzyme OTUB1 promotes cancer cell immunosuppression via preventing ER-associated degradation of immune checkpoint protein PD-L1. Cell Death Differ.

[18] Liu C, Zhou S, Tang W (2024). USP14 promotes the cancer stem-like cell properties of OSCC via promoting SOX2 deubiquitination. Oral Dis.

[19] Tang Z, Jiang W, Mao M, Zhao J, Chen J, Cheng N (2021). Deubiquitinase USP35 modulates ferroptosis in lung cancer via targeting ferroportin. Clin Transl Med.

[20] Song J, Liu T, Yin Y, Zhao W, Lin Z, Yin Y (2021). The deubiquitinase OTUD1 enhances iron transport and potentiates host antitumor immunity. EMBO Rep.

[21] Ren Y, Feng M, Hao X, Liu X, Li J, Li P (2023). USP48 Stabilizes Gasdermin E to Promote Pyroptosis in Cancer. Cancer Res.

[22] Di M, Miao J, Pan Q, Wu Z, Chen B, Wang M (2022). OTUD4-mediated GSDME deubiquitination enhances radiosensitivity in nasopharyngeal carcinoma by inducing pyroptosis. J Exp Clin Cancer Res.

[23] Wang Y, Yang L, Zhang X, Cui W, Liu Y, Sun QR (2019). Epigenetic regulation of ferroptosis by H2B monoubiquitination and p53. EMBO Rep.

[24] Guan X, Wang Y, Yu W, Wei Y, Lu Y, Dai E (2024). Blocking Ubiquitin-Specific Protease 7 Induces Ferroptosis in Gastric Cancer via Targeting Stearoyl-CoA Desaturase. Adv Sci (Weinh).

[25] Wang R, Wang Y, Liu X, Liu M, Sun L, Pan X (2023). Anastasis enhances metastasis and chemoresistance of colorectal cancer cells through upregulating cIAP2/NFκB signaling. Cell Death Dis.

[26] Liu J, Uematsu H, Tsuchida N, Ikeda MA (2009). Association of caspase-8 mutation with chemoresistance to cisplatin in HOC313 head and neck squamous cell carcinoma cells. Biochem Biophys Res Commun.

[27] Li X, Zhao X, Su X, Wen J, Yang S, Qin Y (2024). IQGAP1 overexpression attenuates chemosensitivity through YAP-mediated ferroptosis inhibition in esophageal squamous cell cancer cells. Arch Biochem Biophys.

[28] Zhao G, Liang J, Shan G, Gu J, Xu F, Lu C (2023). KLF11 regulates lung adenocarcinoma ferroptosis and chemosensitivity by suppressing GPX4. Commun Biol.

[29] Roh JL, Kim EH, Jang HJ, Park JY, Shin D (2016). Induction of ferroptotic cell death for overcoming cisplatin resistance of head and neck cancer. Cancer Lett.

[30] Lin H, Tison K, Du Y, Kirchhoff P, Kim C, Wang W (2024). Itaconate transporter SLC13A3 impairs tumor immunity via endowing ferroptosis resistance. Cancer Cell.

[31] Tang D, Chen X, Kang R, Kroemer G (2021). Ferroptosis: molecular mechanisms and health implications. Cell Res.

[32] Latunde-Dada GO (2017). Ferroptosis: Role of lipid peroxidation, iron and ferritinophagy. Biochim Biophys Acta Gen Subj.

[33] Morales M, Xue X (2021). Targeting iron metabolism in cancer therapy. Theranostics.

[34] Puig S, Ramos-Alonso L, Romero AM, Martinez-Pastor MT (2017). The elemental role of iron in DNA synthesis and repair. Metallomics.

[35] Henning Y, Blind US, Larafa S, Matschke J, Fandrey J (2022). Hypoxia aggravates ferroptosis in RPE cells by promoting the Fenton reaction. Cell Death Dis.

[36] Pantopoulos K, Porwal SK, Tartakoff A, Devireddy L (2012). Mechanisms of mammalian iron homeostasis. Biochemistry.

[37] Wang H, Shi H, Rajan M, Canarie ER, Hong S, Simoneschi D (2020). FBXL5 Regulates IRP2 Stability in Iron Homeostasis via an Oxygen-Responsive [2Fe2S] Cluster. Mol Cell.

[38] Geng N, Shi BJ, Li SL, Zhong ZY, Li YC, Xua WL (2018). Knockdown of ferroportin accelerates erastin-induced ferroptosis in neuroblastoma cells. Eur Rev Med Pharmacol Sci.

[39] Babu KR, Muckenthaler MU (2016). miR-20a regulates expression of the iron exporter ferroportin in lung cancer. J Mol Med (Berl).

[40] Rychtarcikova Z, Lettlova S, Tomkova V, Korenkova V, Langerova L, Simonova E (2017). Tumor-initiating cells of breast and prostate origin show alterations in the expression of genes related to iron metabolism. Oncotarget.

[41] Zhang Q, Chen C, Zou X, Wu W, Di Y, Li N (2024). Iron promotes ovarian cancer malignancy and advances platinum resistance by enhancing DNA repair via FTH1/FTL/POLQ/RAD51 axis. Cell Death Dis.

[42] Mancias JD, Wang X, Gygi SP, Harper JW, Kimmelman AC (2014). Quantitative proteomics identifies NCOA4 as the cargo receptor mediating ferritinophagy. Nature.

[43] Xu D, Shan B, Sun H, Xiao J, Zhu K, Xie X (2016). USP14 regulates autophagy by suppressing K63 ubiquitination of Beclin 1. Genes Dev.

[44] Tsai Y, Xia C, Sun Z (2020). The Inhibitory Effect of 6-Gingerol on Ubiquitin-Specific Peptidase 14 Enhances Autophagy-Dependent Ferroptosis and Anti-Tumor in vivo and in vitro. Front Pharmacol.

[45] Banjac A, Perisic T, Sato H, Seiler A, Bannai S, Weiss N (2008). The cystine/cysteine cycle: a redox cycle regulating susceptibility versus resistance to cell death. Oncogene.

[46] Koppula P, Zhuang L, Gan B (2021). Cystine transporter SLC7A11/xCT in cancer: ferroptosis, nutrient dependency, and cancer therapy. Protein Cell.

[47] Xu X, Zhang X, Wei C, Zheng D, Lu X, Yang Y (2020). Targeting SLC7A11 specifically suppresses the progression of colorectal cancer stem cells via inducing ferroptosis. Eur J Pharm Sci.

[48] Zhang B, Bao W, Zhang S, Chen B, Zhou X, Zhao J (2022). LncRNA HEPFAL accelerates ferroptosis in hepatocellular carcinoma by regulating SLC7A11 ubiquitination. Cell Death Dis.

[49] Zhou Q, Yu H, Chen Y, Ren J, Lu Y, Sun Y (2024). The CRL3(KCTD10) ubiquitin ligase-USP18 axis coordinately regulates cystine uptake and ferroptosis by modulating SLC7A11. Proc Natl Acad Sci U S A.

[50] Liu X, Ma Z, Jing X, Wang G, Zhao L, Zhao X (2024). The deubiquitinase OTUD5 stabilizes SLC7A11 to promote progression and reduce paclitaxel sensitivity in triple-negative breast cancer. Cancer Lett.

[51] Liu J, Luo Y, Chen S, Wang G, Jin W, Jiang W (2024). Deubiquitylase USP52 Promotes Bladder Cancer Progression by Modulating Ferroptosis through Stabilizing SLC7A11/xCT. Adv Sci (Weinh).

[52] Feng H, Stockwell BR (2018). Unsolved mysteries: How does lipid peroxidation cause ferroptosis?. PLoS Biol.

[53] Zhang Y, Tan H, Daniels JD, Zandkarimi F, Liu H, Brown LM (2019). Imidazole Ketone Erastin Induces Ferroptosis and Slows Tumor Growth in a Mouse Lymphoma Model. Cell Chem Biol.

[54] Ayob AZ, Ramasamy TS (2018). Cancer stem cells as key drivers of tumour progression. J Biomed Sci.

[55] Wang Z, Ouyang L, Liu N, Li T, Yan B, Mao C (2023). The DUBA-SLC7A11-c-Myc axis is critical for stemness and ferroptosis. Oncogene.

[56] Zhong Q, Xiao X, Qiu Y, Xu Z, Chen C, Chong B (2023). Protein posttranslational modifications in health and diseases: Functions, regulatory mechanisms, and therapeutic implications. MedComm (2020).

[57] Tang J, Long G, Xiao D, Liu S, Xiao L, Zhou L (2023). ATR-dependent ubiquitin-specific protease 20 phosphorylation confers oxaliplatin and ferroptosis resistance. MedComm (2020).

[58] Gao C, Xiao F, Zhang L, Sun Y, Wang L, Liu X (2022). SENP1 inhibition suppresses the growth of lung cancer cells through activation of A20-mediated ferroptosis. Ann Transl Med.

[59] Wang Y, Hong Z, Song J, Zhong P, Lin L (2023). METTL3 promotes drug resistance to oxaliplatin in gastric cancer cells through DNA repair pathway. Front Pharmacol.

[60] Yan B, Guo J, Wang Z, Ning J, Wang H, Shu L (2023). The ubiquitin-specific protease 5 mediated deubiquitination of LSH links metabolic regulation of ferroptosis to hepatocellular carcinoma progression. MedComm (2020).

[61] Fu D, Wang C, Yu L, Yu R (2021). Induction of ferroptosis by ATF3 elevation alleviates cisplatin resistance in gastric cancer by restraining Nrf2/Keap1/xCT signaling. Cell Mol Biol Lett.

[62] Wang L, Liu Y, Du T, Yang H, Lei L, Guo M (2020). ATF3 promotes erastin-induced ferroptosis by suppressing system Xc(). Cell Death Differ.

[63] Li Z, Tian Y, Zong H, Wang X, Li D, Keranmu A (2024). Deubiquitinating enzyme OTUD4 stabilizes RBM47 to induce ATF3 transcription: a novel mechanism underlying the restrained malignant properties of ccRCC cells. Apoptosis.

[64] Yang X, Qian K (2017). Protein O-GlcNAcylation: emerging mechanisms and functions. Nat Rev Mol Cell Biol.

[65] Yu F, Zhang Q, Liu H, Liu J, Yang S, Luo X (2022). Dynamic O-GlcNAcylation coordinates ferritinophagy and mitophagy to activate ferroptosis. Cell Discov.

[66] Chen Y, Zhu G, Liu Y, Wu Q, Zhang X, Bian Z (2019). O-GlcNAcylated c-Jun antagonizes ferroptosis via inhibiting GSH synthesis in liver cancer. Cell Signal.

[67] Zhu G, Murshed A, Li H, Ma J, Zhen N, Ding M (2021). O-GlcNAcylation enhances sensitivity to RSL3-induced ferroptosis via the YAP/TFRC pathway in liver cancer. Cell Death Discov.

[68] Tang J, Long G, Hu K, Xiao D, Liu S, Xiao L (2023). Targeting USP8 Inhibits O-GlcNAcylation of SLC7A11 to Promote Ferroptosis of Hepatocellular Carcinoma via Stabilization of OGT. Adv Sci (Weinh).

[69] Tang J, Long G, Li X, Zhou L, Zhou Y, Wu Z (2023). The deubiquitinase EIF3H promotes hepatocellular carcinoma progression by stabilizing OGT and inhibiting ferroptosis. Cell Commun Signal.

[70] Ning D, Chen J, Du P, Liu Q, Cheng Q, Li X (2021). The crosstalk network of XIST/miR-424-5p/OGT mediates RAF1 glycosylation and participates in the progression of liver cancer. Liver Int.

[71] Minsky N, Shema E, Field Y, Schuster M, Segal E, Oren M (2008). Monoubiquitinated H2B is associated with the transcribed region of highly expressed genes in human cells. Nat Cell Biol.

[72] Zhang Y, Koppula P, Gan B (2019). Regulation of H2A ubiquitination and SLC7A11 expression by BAP1 and PRC1. Cell Cycle.

[73] Liu T, Jiang L, Tavana O, Gu W (2019). The Deubiquitylase OTUB1 Mediates Ferroptosis via Stabilization of SLC7A11. Cancer Res.

[74] Juang YC, Landry MC, Sanches M, Vittal V, Leung CC, Ceccarelli DF (2012). OTUB1 co-opts Lys48-linked ubiquitin recognition to suppress E2 enzyme function. Mol Cell.

[75] Sun XX, Challagundla KB, Dai MS (2012). Positive regulation of p53 stability and activity by the deubiquitinating enzyme Otubain 1. EMBO J.

[76] Deng H, Jia S, Tang J, Rong F, Xu C, Chen X (2023). SET7 methylates the deubiquitinase OTUB1 at Lys (122) to impair its binding to E2 enzyme UBC13 and relieve its suppressive role on ferroptosis. J Biol Chem.

[77] Huang S, Zhang Q, Zhao M, Wang X, Zhang Y, Gan B (2023). The deubiquitinase ZRANB1 is an E3 ubiquitin ligase for SLC7A11 and regulates ferroptotic resistance. J Cell Biol.

[78] Wertz IE, O'Rourke KM, Zhou H, Eby M, Aravind L, Seshagiri S (2004). De-ubiquitination and ubiquitin ligase domains of A20 downregulate NF-kappaB signalling. Nature.

[79] Pedrera L, Espiritu RA, Ros U, Weber J, Schmitt A, Stroh J (2021). Ferroptotic pores induce Ca(2+) fluxes and ESCRT-III activation to modulate cell death kinetics. Cell Death Differ.

[80] Maher P, van Leyen K, Dey PN, Honrath B, Dolga A, Methner A (2018). The role of Ca(2+) in cell death caused by oxidative glutamate toxicity and ferroptosis. Cell Calcium.

[81] Duan J, Huang D, Liu C, Lv Y, Zhang L, Chang F (2023). USP11-mediated LSH deubiquitination inhibits ferroptosis in colorectal cancer through epigenetic activation of CYP24A1. Cell Death Dis.

[82] Fox EJ (2004). Mechanism of action of mitoxantrone. Neurology.

[83] Burkhart RA, Peng Y, Norris ZA, Tholey RM, Talbott VA, Liang Q (2013). Mitoxantrone targets human ubiquitin-specific peptidase 11 (USP11) and is a potent inhibitor of pancreatic cancer cell survival. Mol Cancer Res.

[84] Magtanong L, Ko PJ, To M, Cao JY, Forcina GC, Tarangelo A (2019). Exogenous Monounsaturated Fatty Acids Promote a Ferroptosis-Resistant Cell State. Cell Chem Biol.

[85] Pope LE, Dixon SJ (2023). Regulation of ferroptosis by lipid metabolism. Trends Cell Biol.

[86] Yi J, Zhu J, Wu J, Thompson CB, Jiang X (2020). Oncogenic activation of PI3K-AKT-mTOR signaling suppresses ferroptosis via SREBP-mediated lipogenesis. Proc Natl Acad Sci U S A.

[87] Sen U, Coleman C, Sen T (2023). Stearoyl coenzyme A desaturase-1: multitasker in cancer, metabolism, and ferroptosis. Trends Cancer.

[88] Nagao K, Murakami A, Umeda M (2019). Structure and Function of Δ9-Fatty Acid Desaturase. Chem Pharm Bull (Tokyo).

[89] Tesfay L, Paul BT, Konstorum A, Deng Z, Cox AO, Lee J (2019). Stearoyl-CoA Desaturase 1 Protects Ovarian Cancer Cells from Ferroptotic Cell Death. Cancer Res.

[90] Tao R, Liu Z, Zhang Z, Zhang Z (2024). USP3 promotes cisplatin resistance in non-small cell lung cancer cells by suppressing ACOT7-regulated ferroptosis. Anticancer Drugs.

[91] Doll S, Proneth B, Tyurina YY, Panzilius E, Kobayashi S, Ingold I (2017). ACSL4 dictates ferroptosis sensitivity by shaping cellular lipid composition. Nat Chem Biol.

[92] Huang Q, Ru Y, Luo Y, Luo X, Liu D, Ma Y (2024). Identification of a targeted ACSL4 inhibitor to treat ferroptosis-related diseases. Sci Adv.

[93] Dai Y, Chen Y, Mo D, Jin R, Huang Y, Zhang L (2023). Inhibition of ACSL4 ameliorates tubular ferroptotic cell death and protects against fibrotic kidney disease. Commun Biol.

[94] Cui Z, Sun H, Gao Z, Li C, Xiao T, Bian Y (2024). TRIM21/USP15 balances ACSL4 stability and the imatinib resistance of gastrointestinal stromal tumors. Br J Cancer.

[95] Delvaux M, Hagué P, Craciun L, Wozniak A, Demetter P, Schöffski P (2022). Ferroptosis Induction and YAP Inhibition as New Therapeutic Targets in Gastrointestinal Stromal Tumors (GISTs). Cancers (Basel).

[96] Li J, Liu J, Zhou Z, Wu R, Chen X, Yu C (2023). Tumor-specific GPX4 degradation enhances ferroptosis-initiated antitumor immune response in mouse models of pancreatic cancer. Sci Transl Med.

[97] Li H, Sun Y, Yao Y, Ke S, Zhang N, Xiong W (2024). USP8-governed GPX4 homeostasis orchestrates ferroptosis and cancer immunotherapy. Proc Natl Acad Sci U S A.

[98] Wu J, Liu C, Wang T, Liu H, Wei B (2023). Deubiquitinase inhibitor PR-619 potentiates colon cancer immunotherapy by inducing ferroptosis. Immunology.

[99] Chu LK, Cao X, Wan L, Diao Q, Zhu Y, Kan Y (2023). Autophagy of OTUD5 destabilizes GPX4 to confer ferroptosis-dependent kidney injury. Nat Commun.

[100] Liu L, Pang J, Qin D, Li R, Zou D, Chi K (2023). Deubiquitinase OTUD5 as a Novel Protector against 4-HNE-Triggered Ferroptosis in Myocardial Ischemia/Reperfusion Injury. Adv Sci (Weinh).

[101] Zhang J, Tian T, Li X, Xu K, Lu Y, Li X (2025). p53 inhibits OTUD5 transcription to promote GPX4 degradation and induce ferroptosis in gastric cancer. Clin Transl Med.

[102] Gong Y, Li R, Zhang R, Jia L (2025). USP2 reversed cisplatin resistance through p53-mediated ferroptosis in NSCLC. BMC Med Genomics.

[103] Xu R, Wang W, Zhang W (2023). Ferroptosis and the bidirectional regulatory factor p53. Cell Death Discov.

[104] Zhang Y, Shi H, Wang Y, Liu W, Li G, Li D (2025). Noscapine derivative 428 suppresses ferroptosis through targeting GPX4. Redox biol.

[105] Wang Z, Xia Y, Wang Y, Zhu R, Li H, Liu Y (2023). The E3 ligase TRIM26 suppresses ferroptosis through catalyzing K63-linked ubiquitination of GPX4 in glioma. Cell Death Dis.

[106] Li D, Wang Y, Dong C, Chen T, Dong A, Ren J (2023). CST1 inhibits ferroptosis and promotes gastric cancer metastasis by regulating GPX4 protein stability via OTUB1. Oncogene.

[107] Tang J, Long G, Xiao L, Zhou L (2023). USP8 positively regulates hepatocellular carcinoma tumorigenesis and confers ferroptosis resistance through beta-catenin stabilization. Cell Death Dis.

[108] Zhang Z, Zhou D, Qiu X, Xia F, Li X (2025). N6-methyladenosine-mediated EIF3H promotes anaplastic thyroid cancer progression and ferroptosis resistance by stabilizing β-catenin. Free Radic Biol Med.

[109] Wang Y, Zheng L, Shang W, Yang Z, Li T, Liu F (2022). Wnt/beta-catenin signaling confers ferroptosis resistance by targeting GPX4 in gastric cancer. Cell Death Differ.

[110] Ma J, Li Z, Xu J, Lai J, Zhao J, Ma L (2024). PRDM1 promotes the ferroptosis and immune escape of thyroid cancer by regulating USP15-mediated SELENBP1 deubiquitination. J Endocrinol Invest.

[111] Zhao W, Nikolic-Paterson DJ, Li K, Li Y, Wang Y, Chen X (2024). Selenium binding protein 1 protects renal tubular epithelial cells from ferroptosis by upregulating glutathione peroxidase 4. Chem Biol Interact.

[112] Zeng F, Chen X, Deng G (2022). The anti-ferroptotic role of FSP1: current molecular mechanism and therapeutic approach. Mol Biomed.

[113] Cheu JW, Lee D, Li Q, Goh CC, Bao MH, Yuen VW (2023). Ferroptosis Suppressor Protein 1 Inhibition Promotes Tumor Ferroptosis and Anti-tumor Immune Responses in Liver Cancer. Cell Mol Gastroenterol Hepatol.

[114] Xie J, Ma C, Zhao S, Wu D, Zhang P, Tang Q Deubiquitination by USP7 Stabilizes JunD and Activates AIFM2 (FSP1) to Inhibit Ferroptosis in Melanoma. J Invest Dermatol. 2025: S0022-202X(25)00381-1.

[115] Li W, Yu S, Liu T, Kim JH, Blank V, Li H (2008). Heterodimerization with small Maf proteins enhances nuclear retention of Nrf2 via masking the NESzip motif. Biochim Biophys Acta.

[116] Dodson M, Castro-Portuguez R, Zhang DD (2019). NRF2 plays a critical role in mitigating lipid peroxidation and ferroptosis. Redox Biol.

[117] Feng L, Zhao K, Sun L, Yin X, Zhang J, Liu C (2021). SLC7A11 regulated by NRF2 modulates esophageal squamous cell carcinoma radiosensitivity by inhibiting ferroptosis. J Transl Med.

[118] Liu J, Huang C, Liu J, Meng C, Gu Q, Du X (2023). Nrf2 and its dependent autophagy activation cooperatively counteract ferroptosis to alleviate acute liver injury. Pharmacol Res.

[119] Wang S, Wang T, Zhang X, Cheng S, Chen C, Yang G (2023). The deubiquitylating enzyme USP35 restricts regulated cell death to promote survival of renal clear cell carcinoma. Cell Death Differ.

[120] Ding Y, Gao J, Chen J, Ren J, Jiang J, Zhang Z (2024). BUB1b impairs chemotherapy sensitivity via resistance to ferroptosis in lung adenocarcinoma. Cell Death Dis.

[121] Tan Y, Chen Q, Li X, Zeng Z, Xiong W, Li G (2021). Correction to: Pyroptosis: a new paradigm of cell death for fighting against cancer. J Exp Clin Cancer Res.

[122] Broz P, Pelegrin P, Shao F (2020). The gasdermins, a protein family executing cell death and inflammation. Nat Rev Immunol.

[123] Chen X, He WT, Hu L, Li J, Fang Y, Wang X (2016). Pyroptosis is driven by non-selective gasdermin-D pore and its morphology is different from MLKL channel-mediated necroptosis. Cell Res.

[124] Miao EA, Rajan JV, Aderem A (2011). Caspase-1-induced pyroptotic cell death. Immunol Rev.

[125] Kesavardhana S, Malireddi RKS, Kanneganti TD (2020). Caspases in Cell Death, Inflammation, and Pyroptosis. Annu Rev Immunol.

[126] Xuzhang W, Lu T, Jin W, Yu Y, Li Z, Shen L (2024). Cisplatin-induced Pyroptosis Enhances the Efficacy of PD-L1 Inhibitor in Small-Cell Lung Cancer via GSDME/IL12/CD4Tem Axis. Int J Biol Sci.

[127] Wang Y, Yin B, Li D, Wang G, Han X, Sun X (2018). GSDME mediates caspase-3-dependent pyroptosis in gastric cancer. Biochem Biophys Res Commun.

[128] Yu J, Li S, Qi J, Chen Z, Wu Y, Guo J (2019). Cleavage of GSDME by caspase-3 determines lobaplatin-induced pyroptosis in colon cancer cells. Cell Death Dis.

[129] Wang Y, Gao W, Shi X, Ding J, Liu W, He H (2017). Chemotherapy drugs induce pyroptosis through caspase-3 cleavage of a gasdermin. Nature.

[130] Yokomizo K, Harada Y, Kijima K, Shinmura K, Sakata M, Sakuraba K (2012). Methylation of the DFNA5 gene is frequently detected in colorectal cancer. Anticancer Res.

[131] Croes L, Beyens M, Fransen E, Ibrahim J, Vanden Berghe W, Suls A (2018). Large-scale analysis of DFNA5 methylation reveals its potential as biomarker for breast cancer. Clin Epigenetics.

[132] Arimoto KI, Miyauchi S, Troutman TD, Zhang Y, Liu M, Stoner SA (2023). Expansion of interferon inducible gene pool via USP18 inhibition promotes cancer cell pyroptosis. Nat Commun.

[133] Zhang Z, Zhang Y, Xia S, Kong Q, Li S, Liu X (2020). Gasdermin E suppresses tumour growth by activating anti-tumour immunity. Nature.

[134] Hou X, Xia J, Feng Y, Cui L, Yang Y, Yang P (2021). USP47-Mediated Deubiquitination and Stabilization of TCEA3 Attenuates Pyroptosis and Apoptosis of Colorectal Cancer Cells Induced by Chemotherapeutic Doxorubicin. Front Pharmacol.

[135] Zhou B, Zhang JY, Liu XS, Chen HZ, Ai YL, Cheng K (2018). Tom20 senses iron-activated ROS signaling to promote melanoma cell pyroptosis. Cell Res.

[136] Hsu CG, Chavez CL, Zhang C, Sowden M, Yan C, Berk BC (2022). The lipid peroxidation product 4-hydroxynonenal inhibits NLRP3 inflammasome activation and macrophage pyroptosis. Cell Death Differ.

[137] Wang H, Shu L, Lv C, Liu N, Long Y, Peng X (2024). BRCC36 Deubiquitinates HMGCR to Regulate the Interplay Between Ferroptosis and Pyroptosis. Adv Sci (Weinh).

[138] Lauinger L, Li J, Shostak A, Cemel IA, Ha N, Zhang Y (2017). Thiolutin is a zinc chelator that inhibits the Rpn11 and other JAMM metalloproteases. Nat Chem Biol.

[139] Hou J, Li T, Hsu JM, Zhang X, Hung MC (2023). Gasdermins and cancers. Semin Immunol.

[140] Wilson TR, Johnston PG, Longley DB (2009). Anti-apoptotic mechanisms of drug resistance in cancer. Curr Cancer Drug Targets.

[141] Wu J, Chen X, Zhang J, Chen J, Wang Y, Wei T (2021). Tachyplesin induces apoptosis in non-small cell lung cancer cells and enhances the chemosensitivity of A549/DDP cells to cisplatin by activating Fas and necroptosis pathway. Chem Biol Drug Des.

[142] Zheng Y, Wang Y, Lu Z, Wan J, Jiang L, Song D (2023). PGAM1 Inhibition Promotes HCC Ferroptosis and Synergizes with Anti-PD-1 Immunotherapy. Adv Sci (Weinh).

[143] Zhou Y, Zhang W, Wang B, Wang P, Li D, Cao T (2024). Mitochondria-targeted photodynamic therapy triggers GSDME-mediated pyroptosis and sensitizes anti-PD-1 therapy in colorectal cancer. J Immunother Cancer.

[144] Liao Y, Liang J, Wang Y, Li A, Liu W, Zhong B (2024). Target deubiquitinase OTUB1 as a therapeatic strategy for BLCA via β-catenin/necroptosis signal pathway. Int J Biol Sci.

[145] Roedig J, Kowald L, Juretschke T, Karlowitz R, Ahangarian Abhari B, Roedig H (2021). USP22 controls necroptosis by regulating receptor-interacting protein kinase 3 ubiquitination. EMBO Rep.

[146] Douglas T, Saleh M (2019). Post-translational Modification of OTULIN Regulates Ubiquitin Dynamics and Cell Death. Cell Rep.

[147] Onizawa M, Oshima S, Schulze-Topphoff U, Oses-Prieto JA, Lu T, Tavares R (2015). Erratum: The ubiquitin-modifying enzyme A20 restricts ubiquitination of the kinase RIPK3 and protects cells from necroptosis. Nat Immunol.

[148] Kim JY, Lee DM, Woo HG, Kim KD, Lee HJ, Kwon Y-J (2019). RNAi screening-based identification of USP10 as a novel regulator of paraptosis. Sci Rep.

[149] Weber A, Heinlein M, Dengjel J, Alber C, Singh PK, Häcker G (2016). The deubiquitinase Usp27x stabilizes the BH3-only protein Bim and enhances apoptosis. EMBO Rep.

[150] Jeong M, Lee EW, Seong D, Seo J, Kim JH, Grootjans S (2017). USP8 suppresses death receptor-mediated apoptosis by enhancing FLIP(L) stability. Oncogene.

[151] Corno C, D'Arcy P, Bagnoli M, Paolini B, Costantino M, Carenini N (2022). The deubiquitinase USP8 regulates ovarian cancer cell response to cisplatin by suppressing apoptosis. Front Cell Dev Biol.

[152] Pan T, Li X, Li Y, Tao Z, Yao H, Wu Y (2021). USP7 inhibition induces apoptosis in glioblastoma by enhancing ubiquitination of ARF4. Cancer Cell Int.

[153] Zhang X, Zhang H, Xu C, Li X, Li M, Wu X (2019). Ubiquitination of RIPK1 suppresses programmed cell death by regulating RIPK1 kinase activation during embryogenesis. Nat Commun.

[154] Yao K, Shi Z, Zhao F, Tan C, Zhang Y, Fan H (2025). RIPK1 in necroptosis and recent progress in related pharmaceutics. Front Immunol.

[155] Jiang M, Qi L, Li L, Li Y (2020). The caspase-3/GSDME signal pathway as a switch between apoptosis and pyroptosis in cancer. Cell Death Discov.

[156] Kracikova M, Akiri G, George A, Sachidanandam R, Aaronson SA (2013). A threshold mechanism mediates p53 cell fate decision between growth arrest and apoptosis. Cell Death Differ.

[157] Kim J, Alavi Naini F, Sun Y, Ma L (2018). Ubiquitin-specific peptidase 2a (USP2a) deubiquitinates and stabilizes beta-catenin. Am J Cancer Res.

[158] Wu C, Luo K, Zhao F, Yin P, Song Y, Deng M (2018). USP20 positively regulates tumorigenesis and chemoresistance through β-catenin stabilization. Cell Death Differ.

[159] Shi J, Liu Y, Xu X, Zhang W, Yu T, Jia J (2015). Deubiquitinase USP47/UBP64E regulates β-catenin ubiquitination and degradation and plays a positive role in Wnt signaling. Mol Cell Biol.

[160] Sahtoe DD, Sixma TK (2015). Layers of DUB regulation. Trends Biochem Sci.

[161] Saldana M, VanderVorst K, Berg AL, Lee H, Carraway KL (2019). Otubain 1: a non-canonical deubiquitinase with an emerging role in cancer. Endocr Relat Cancer.

[162] Rowinsky EK, Paner A, Berdeja JG, Paba-Prada C, Venugopal P, Porkka K (2020). Phase 1 study of the protein deubiquitinase inhibitor VLX1570 in patients with relapsed and/or refractory multiple myeloma. Invest New Drugs.

[163] Chan WC, Liu X, Magin RS, Girardi NM, Ficarro SB, Hu W (2023). Accelerating inhibitor discovery for deubiquitinating enzymes. Nat Commun.

[164] Goracci L, Desantis J, Valeri A, Castellani B, Eleuteri M, Cruciani G (2020). Understanding the Metabolism of Proteolysis Targeting Chimeras (PROTACs): The Next Step toward Pharmaceutical Applications. J Med Chem.

[165] Oleksiewicz U, Tomczak K, Woropaj J, Markowska M, Stępniak P, Shah PK (2015). Computational characterisation of cancer molecular profiles derived using next generation sequencing. Contemp Oncol (Pozn).

[166] Dai E, Han L, Liu J, Xie Y, Kroemer G, Klionsky DJ (2020). Autophagy-dependent ferroptosis drives tumor-associated macrophage polarization via release and uptake of oncogenic KRAS protein. Autophagy.

[167] Tan G, Huang C, Chen J, Zhi F (2020). HMGB1 released from GSDME-mediated pyroptotic epithelial cells participates in the tumorigenesis of colitis-associated colorectal cancer through the ERK1/2 pathway. J Hematol Oncol.

[168] Dai E, Han L, Liu J, Xie Y, Zeh HJ, Kang R (2020). Ferroptotic damage promotes pancreatic tumorigenesis through a TMEM173/STING-dependent DNA sensor pathway. Nat Commun.

[169] Kim R, Hashimoto A, Markosyan N, Tyurin VA, Tyurina YY, Kar G (2022). Ferroptosis of tumour neutrophils causes immune suppression in cancer. Nature.

[170] Ma X, Xiao L, Liu L, Ye L, Su P, Bi E (2021). CD36-mediated ferroptosis dampens intratumoral CD8(+) T cell effector function and impairs their antitumor ability. Cell Metab.

[171] Han L, Bai L, Qu C, Dai E, Liu J, Kang R (2021). PPARG-mediated ferroptosis in dendritic cells limits antitumor immunity. Biochem Biophys Res Commun.

[172] Wang M, Prachyathipsakul T, Wisniewski CA, Xiong C, Goel S, Goel HL (2024). Therapeutic induction of ferroptosis in tumors using PD-L1 targeting antibody nanogel conjugates. Cell Chem Biol.

[173] Zaffaroni N, Beretta GL (2021). Nanoparticles for Ferroptosis Therapy in Cancer. Pharmaceutics.

[174] Wiernicki B, Maschalidi S, Pinney J, Adjemian S, Vanden Berghe T, Ravichandran KS (2022). Cancer cells dying from ferroptosis impede dendritic cell-mediated anti-tumor immunity. Nat Commun.

[175] Jin KT, Du WL, Lan HR, Liu YY, Mao CS, Du JL (2021). Development of humanized mouse with patient-derived xenografts for cancer immunotherapy studies: A comprehensive review. Cancer Sci.

